# Glis1 regulates metabolic programming by promoting H3K18 lactylation to alleviate neuronal senescence following spinal cord injury

**DOI:** 10.1016/j.gendis.2026.102382

**Published:** 2026-07-23

**Authors:** Dachuan Li, Xiao Lu, Zhaoyang Gong, Jianwei Wu, Yuxuan Zhang, Jiongdong Wu, Chenpei Xu, Xingyu Guo, Guangyu Xu, Haocheng Xu, Shuo Yang, Xinlei Xia, Feizhou Lu, Jianyuan Jiang, Shihao Lu, Junzhe Sheng, Xiaosheng Ma, Wei Zhu, Fei Zou, Chi Sun, Xiaochuan Gu, Yunzhi Guan

**Affiliations:** a Department of Orthopedics, Huashan Hospital, Fudan University, Shanghai 200040, China; b Department of Orthopedics, Changhai Hospital, Navy Medical University, Shanghai 200433, China; c Department of Spinal Surgery, Changzheng Hospital, Navy Medical University, Shanghai 200003, China

**Keywords:** Cellular senescence, Histone proteins, Lactylation, Metabolic reprogramming, SCI

## Abstract

Neuronal senescence is a significant factor impeding spinal cord injury (SCI) repair. Research indicates that Glis1, a Kruppel-like zinc finger transcription factor, is pivotal in reprogramming senescent cells and enhancing genomic stability via lactylation. Histone lactylation, an epigenetic modification using lactate as a substrate, becomes prominent post-SCI due to increased neuronal lactate levels. However, the mechanisms linking neuronal senescence and histone lactylation following SCI remain poorly understood. This study elucidates the roles of Glis1, H3K18 lactylation, and neuronal senescence through comprehensive *in vivo* and *in vitro* investigations. The findings reveal a post-SCI rise in neuronal lactate levels, accompanied by cellular senescence. Furthermore, upregulation of Glis1 attenuates senescence and promotes histone lactylation. The downstream target secretogranin II (SCG2), identified via ChIP-seq and RNA-seq, was found to exert negative feedback on glycolytic metabolism and lactylation, thereby suppressing neuronal senescence. These results unveil a novel regulatory mechanism of Glis1 in neuronal senescence following SCI through the p300/H3K18la/SCG2 axis, offering a new therapeutic target for spinal cord repair.

## Introduction

Spinal cord injury (SCI) is a debilitating condition often encountered in spinal surgery, leading to significant functional impairments below the injured area and affecting millions worldwide. The challenge of injured spinal cord repair is largely due to its complex pathophysiology, which encompasses multiple intertwined processes.^1^ Despite extensive research and diverse regenerative therapies, effective injured spinal cord repair remains a contentious issue, and efforts continue to seek strategies to prevent or mitigate its devastating impact.^2^ This underscores the need for deeper insights into the underlying mechanisms to enhance outcomes for patients with SCI. SCI induces an ischemic and hypoxic microenvironment in the metabolically active spinal cord, disrupting the interplay between glycolysis and oxidative phosphorylation, leading to lactic acid accumulation alongside neural inflammation.^3^^,^^4^ Some studies further indicate that hypoxia at the injury site exacerbates glycolysis and lactate buildup, compromising the catabolic balance and diminishing neuronal repair and regenerative capacity, which may aggravate injury.^5^ Although lactate, a byproduct of glycolysis under hypoxic conditions, is recognized as a key metabolic substrate and a potential regulator of cellular functions post-SCI, its exact role in metabolic programming following SCI remains unclear.^6^

Histone lactylation, an epigenetic modification, influences gene transcription by altering histone structure.^6^ When intracellular levels of lactate reach a certain threshold, a “lactate clock” mechanism initiates lysine lactylation through enzymatic processes. In this process, pro-lactylation enzymes transfer lactate to histone lysine residues, activating transcriptional signals interpreted by effector proteins known as “readers,” which indirectly regulate gene expression.^7^ Studies suggest that histone lactylation plays a pivotal role in coordinating various physiological processes in organisms.^8^ However, the specific functions and regulatory mechanisms of histone lactylation in the context of SCI remain largely unexplored.

Neurons are the primary structural and functional units of the nervous system, playing a critical role in SCI pathophysiology.^9^ Cellular senescence refers to a state of irreversible cell cycle arrest characterized by persistent inflammation and the expression of senescence-associated secretory phenotypes (SASPs). It occurs as either physiological senescence, driven by telomere shortening (replicative senescence), or accelerated senescence, induced by external stressors (post-acute injury senescence).^10^ Accelerated senescence significantly contributes to various diseases, with evidence showing the presence of senescent neurons around the lesion site following SCI, where the neuroinflammatory response is closely linked to SASP activity.^11^^,^^12^ Moreover, targeting senescent neuronal cells—specifically, inhibiting neuronal senescence—has been found to enhance the post-injury repair microenvironment.^13^

Glis1, a Kruppel-like zinc-finger transcription factor, acts as both an activator and repressor of gene transcription, regulating numerous physiological and pathological processes.^14^ By promoting H3K18 lactylation, Glis1 enhances genomic stability, suggesting its potential significance in SCI research, particularly concerning lactylation modifications. However, the precise role of lactate and histone lactylation and their subsequent effects on metabolic regulation post-SCI remain inadequately understood. This study aims to investigate Glis1 as a “lactate-responsive” molecule, exploring for the first time its role in modulating neuronal senescence under elevated lactate conditions following SCI. The research focuses on elucidating whether Glis1's molecular mechanism involves histone lactylation modification and the regulation of metabolic reprogramming.

## Materials and methods

### Animals

Forty-eight-week-old Sprague–Dawley (SD) rats (200–300 g) were obtained from the Animal Experiment Center of authors' affiliation and housed under specific pathogen-free conditions in standard cages (4 per cage). The facility maintained a 12-h light/dark cycle with controlled temperature and humidity. Before starting the experiments, the rats underwent a one-week acclimation period. All procedures adhered to protocols approved by the Animal Protection and Utilization Committee of authors’ affiliation (202408016S).

### SCI and drug treatment

Gene knockdown was performed using adeno-associated virus (AAV) vectors carrying short hairpin RNA (shRNA) targeting Glis1 (sh-Glis1) or a control shRNA (sh-NC) (OBiO Technology, Shanghai) to achieve localized Glis1 depletion *in vivo*.^15^ Rats were anesthetized with an intraperitoneal injection of ketamine (70 mg/kg, Hengrui, China) and xylazine (5 mg/kg, Maclean, China),^16^ placed in a prone position, and underwent laminectomy at T8/T9 to expose the spinal cord. Spinal cord contusion was then induced using a modified impactor (2 mm diameter, 12.5 g weight, 1 cm height).^17^ For the Sham group, the same procedures were performed without contusion, followed by direct suturing after hemostasis. For the SCI + sh-NC and SCI + sh-Glis1 groups, AAV vector microinjections were carried out using a NanoFil microinjection system (Sarasota, FL) post-SCI, following the dosing guidelines of Rong and Li et al.^18^^,^^19^ Each rat received 10 μL of sh-NC or sh-Glis1 solution at a rate of 200 nL/min, with viral vectors (5 × 10^9^ vg; 1 μL) injected at a depth of 1 mm into the lesion site at multiple angles. The Sham and SCI groups received equivalent volumes of DMSO. Throughout the study, the rats' health, activity, and potential signs of infection were monitored daily. Postoperative care included analgesic treatment with meloxicam (1 mg/kg, Boehringer) and antibiotic administration of gentamicin sulfate (4 mg/kg, Huachu) for three days. Bladder function was supported by twice-daily manual bladder expression until voluntary urination was restored. Spinal cord tissues used for experiments were collected from a 5-mm long spinal cord segment centered at the lesion epicenter. This ensures that the tissue samples capture the core pathological changes, including the lesion site and the immediate penumbra.

### Magnetic resonance imaging (MRI) of spinal cord

At 4 weeks after the surgical procedure, rats were anesthetized by intraperitoneal injection and subsequently scanned using a 3.0 T MRI imaging device (Prism, Siemens, Germany). After successful establishment of anesthesia, the rats were positioned on the MRI coil, with their spines aligned to be straight and oriented vertically through the coil. Two orthopedic fellows assessed the MRI images.

### Motor behavior assessment

According to the Basso, Beattie and Bresnahan (BBB) motor score, the motor recovery of rats in each group was evaluated.^20^^,^^21^ The procedure involved permitting the rats to move freely for 5 min within a 90 cm^2^ area while monitoring their hind limb locomotion and coordination. Assessments for the Sham group, SCI group, SCI + sh-NC group, and SCI + sh-Glis1 group were conducted on postoperative days 3, 7, 14, 21, and 28. Assessors were unaware of the experimental group at the time of scoring, and the assessment was repeated three times per rat and recorded immediately. Scores range from 0 to 21, with 0 indicating no motor function and 21 indicating normal motor function.

### Cell culture

The spinal cords of 24-h-old newborn Wistar rats were digested with 0.25% trypsin in a balanced salt solution (BSS) for 15 min at 37 °C, followed by mechanical dissociation to obtain a cell suspension. The cells were then resuspended in DMEM (Gibco, Carlsbad, CA) supplemented with 10% fetal bovine serum and 50 U/mL cytarabine (Invitrogen, Carlsbad, CA) and cultured at 37 °C in a 5% CO_2_ atmosphere. After 7 days, the neurons were collected and purified by shaking at 180 rpm for 6 h at 37 °C. The cells were then examined under a light microscope, revealing healthy neuronal cytoplasm and robustly growing, interconnected protrusions (Fig. S1A). For further experiments, cells were washed three times with phosphate-buffered saline (PBS), passaged once they reached 90% confluence, and subjected to cellular immunofluorescence analysis, confirming the morphological characteristics of spinal cord neurons. To simulate neuronal alterations following SCI *in vitro*, neuronal cells were seeded at a density of approximately 1 × 10^6^/mL 24 h prior to treatment. SCI was induced by exposing neurons to lipopolysaccharide (LPS) (1 μg/mL) for 24 h. Additional treatments included exposure to 8 mM OXAMATE (a glycolysis inhibitor) for 24 h and 5 mM lactic acid for 24 h.^22^^,^^23^ Given that previous studies have demonstrated that neurons express functional Toll-like receptor 4 (TLR4),^24^, ^25^, ^26^, ^27^ this model was utilized to investigate the direct impact of inflammatory stimuli on neuronal senescence.^28^ For gene modulation experiments, si-Glis1, pc-Glis1, pc-SCG2 (secretogranin II), and pc-p300 plasmids (constructed by Genechem Co., Shanghai, China) were transfected into cultured neurons using Lipofectamine 2000 reagent (Invitrogen, Carlsbad, CA).

### Western blot

Neuronal cells and spinal cord tissues were mixed in buffer containing a protease inhibitor (AS1008, Aspen) for 15 s to ensure complete suspension and dispersion, followed by incubation in an ice bath for 30 min. The mixture was then shaken at high speed for 5 s and centrifuged at 13,000×*g* for 10 min at 4 °C. Protein concentration was measured using the BCA Protein Assay Kit (PC0020, Solarbio, Beijing, China). Protein samples underwent denaturation in a boiling water bath at 95–100 °C for 5 min, followed by SDS‒PAGE for separation and transfer onto a PVDF membrane (IPVH00010, Millipore). The PVDF membrane was incubated overnight at 4 °C with a primary antibody diluted in the appropriate diluent, as specified in Table S1. After incubation, the membrane was washed three times with Tris-buffered saline with Tween (5 min for each wash) and then incubated with a secondary antibody for 30 min at room temperature. Protein bands were visualized using an enhanced chemiluminescence (ECL) mixture (BL520B, Biosharp, China) and quantified with ImageJ software. Protein expression was calculated as the ratio of the gray value of the target protein band to the internal reference band's gray value.

### CCK-8

To evaluate neuronal survival, the Cell Counting Kit-8 (CCK-8) (C0038, Beyotime) was utilized following the manufacturer's instructions. In brief, primary neurons were seeded into 96-well plates at a density of 1 × 10^4^ cells per well. Upon completion of the experimental treatments, the culture medium was supplemented with 10 μL of CCK-8 solution, followed by an incubation period of 2 h at 37 °C. The optical density (OD) was subsequently quantified at a wavelength of 450 nm using a microplate reader (DR-200BS, Diatek). The relative cell viability was calculated as a percentage of the control group.

### Histological analysis and neural-like cell assessments

Four weeks post-SCI, rats were deeply anesthetized with ketamine (0.1 mg/g) and sacrificed, and their spinal cord tissues were fixed in 4% paraformaldehyde for 48 h. Tissues were then rinsed under running water for 4 h, dehydrated through a graded series, immersed in xylene, and embedded in paraffin. Sections were cut at a thickness of 7 μm. Paraffin sections were rehydrated and stained with hematoxylin (Solarbio, Beijing, China) for 5 min and eosin for 2 min, followed by observation under a light microscope to assess cellular and extracellular matrix characteristics. Nissl staining was performed by applying Nissl's staining solution (Sigma, 10020818, 71041284) for 5–10 min, followed by differentiation in 1% glacial acetic acid for 2–3 s, washing with distilled water, air drying, clearing in xylene for 5 min, and sealing with neutral tree gum. The number of neurons in the anterior horn of spinal cord sections at identical anatomical levels was evaluated using light microscopy.

To assess fibrosis and collagen content, Masson's trichrome staining (G1006, Servicebio) was applied. Additionally, immunostaining for neurofilament protein (NF) and glial fibrillary acidic protein (GFAP) was used to evaluate neuronal cell numbers and functional status.

### Immunofluorescence assessment

Spinal cord tissue samples were collected as previously described. Neuronal cell samples, after undergoing cell culture treatments, were fixed in 4% paraformaldehyde for 20 min and washed three times with PBS, with each wash lasting 5 min. Sections were incubated with the primary antibodies (diluted with 5% BSA) listed in Table S1 and exposed to the primary antibodies overnight at 4 °C. Following three PBS washes, the cells were incubated with the corresponding secondary antibody at 37 °C for 40 min and then washed three additional times with PBS (5 min per wash). After 4′, 6-Diamidino-2-phenylindole (DAPI) staining, the cells were kept in the dark at room temperature for 20–30 min. Anti-fluorescence quenching sealant was used to preserve the fluorescence, and images were captured using a Nikon ECLIPSE Ci-L microscope (Nikon, Japan). Fluorescence intensity was measured with ImageJ software.

### Luciferase assay

The SCG2 promoter (−1800/+200 bp) of the genomic DNA of SD rats was isolated and inserted into the pGL3 basic vector (ELK Biotechnology). The Glis1 plasmid (pc-Glis1), luciferase reporter plasmid, and control reporter plasmid were transfected into neuronal cells by means of Lipofectamine 2000 (Invitrogen), followed by treatment with the glycolysis inhibitor OXAMATE. The luciferase activity of each group was determined using a dual luciferase reporter assay (RG028, Beyotime) in accordance with the manufacturer's protocol.

### Chromatin immunoprecipitation (ChIP)-PCR

Neuronal cells were washed with PBS. The cells were crosslinked with formaldehyde and lysed by sonication with 500 μL of cell lysate. Centrifugation was performed at 12,000 rpm for 10 min, and the supernatant was transferred to a new EP tube. Antibodies (negative control IgG) were added to magnetic beads and mixed at 4 °C for 30 min. The magnetic beads were adsorbed on a magnetic rack. Five hundred microliters of sample was used as the “Input” and stored at −80 °C. Supernatant (250 μL) was incubated with 5 μL of target antibody or 5 μL of IgG overnight at 4 °C with shaking. Activated magnetic beads were added and incubated at 4 °C for 1 h. The mixture was left to stand, and the supernatant was removed. After elution and reverse cross-linking, DNA purification was performed (A1701A, Takara). Precipitation was performed as ChIP samples, which were analyzed using quantitative PCR (qPCR) and agarose gel electrophoresis.

### Co-immunoprecipitation (Co-IP)

Neuronal cells were lysed by adding IP lysis buffer (AS1004, ASPEN) to ensure complete lysis of the cells. The supernatant was collected by centrifugation at 12,000 rpm for 5 min at 4 °C and incubated overnight at 4 °C with shaking. The IP lysate and 50 μL of ProteinA/G magnetic beads (#1614813, Bio-Rad) were incubated for 30 min at 4 °C with shaking. The precipitate was washed with the lysate and PBS and resuspended in 40 μL of kinase buffer, followed by the addition of 200 μM ATP and the substrate binding reaction, and subjected to Western blotting (WB) analysis as described above.

### Quantitative real-time polymerase chain reaction (qRT–PCR)

For gene expression analysis, total RNA was extracted from neuronal cells using TRIzol reagent (EP013, ELK Biotechnology). The RNA was reverse-transcribed into cDNA, and real-time qPCR was performed using the EnTurbo™ SYBR Green PCR SuperMix kit (ELK Biotechnology, EQ001) on a QuantStudio 6 Flex System (Life Technologies). The relative mRNA expression of the SCG2 gene was calculated using the 2^−ΔΔCt^ method, with the primer sequences provided in Table S2.

### Lactate measurement

Lactate levels were measured using the l-Lactic Acid (L-LA) Content Assay Kit (BC2235, Solarbio) according to the kit instructions. Briefly, 1 mL of Extract I was added to the cells, homogenized, ground and centrifuged, and 0.8 mL of supernatant was added to 0.15 mL of Extract II, which was then centrifuged, diluted 5-fold and mixed with the assay solution. The absorbance was measured at 570 nm using a spectrophotometer (BioTek ELX800, USA), and the lactic acid content was calculated from a standard curve based on the concentration of the standard solution.

### Cell migration assay

To evaluate the paracrine effects of neurons on angiogenesis, primary neurons were subjected to the indicated treatments (control, LPS, LPS + pc-Glis1, and LPS + pc-Glis1+OXAMATE) for 24 h. The culture supernatants were collected, centrifuged at 2000×*g* for 10 min to remove cell debris, and defined as neuronal conditioned medium (CM). For the migration assay, HUVECs were seeded into 6-well plates at a density of approximately 5 × 10^5^ cells per well and cultured until they reached 90% confluence. A linear scratch was created across the cell monolayer using a sterile 200 μL pipette tip. After washing with PBS to remove detached cells, the HUVECs were incubated in the aforementioned neuronal CM supplemented with 1% fetal bovine serum to minimize cell proliferation. Images of the scratch area were captured immediately (0 h) and after 24 h of incubation using an inverted microscope. The wound closure area was analyzed using ImageJ software to calculate the cell migration rate.

### Tube formation assays

To assess capillary-like structure formation, HUVECs were resuspended in the various neuronal CM (collected as described above). The cells were then inoculated onto 96-well plates precoated with Matrigel (354248, Corning) at a density of 1 × 10^4^ cells/well and cultured in an incubator with 5% CO_2_ at 37 °C. After 4 h of incubation, the tube-like networks assembled by HUVECs on the Matrigel surface were observed and photographed using an inverted optical microscope. The number of formed tubes was analyzed and quantified using ImageJ software to evaluate the angiogenic capacity induced by the neuronal paracrine factors.

### Detection of NAD^+^/NADH

The assay was performed using the NAD^+^/NADH Assay Kit (S0175, Beyotime) by adding 200 μL of precooled NAD^+^/NADH extract in an ice bath at a ratio of 200 μL of extract per 1 million cells according to the instructions and gently pipetting to promote cell lysis. Subsequently, centrifugation was carried out at 12,000 rpm and 4 °C for 5–10 min, and the supernatant was retrieved as the sample to be tested. Twenty microliters of the supernatant diluted with NAD^+^/NADH extract was tested in a 96-well plate, and blank control wells, standard wells and sample wells were set up. Ethanol dehydrogenase working solution was added and mixed well. Then, 10 μL of color development solution was added to each well, mixed well and incubated at 37 °C for 20 min. The absorbance at 450 nm was measured, and NAD^+^/NADH was calculated from the standard curve prepared from the standard solution concentrations.

### Mitochondrial oxygen consumption and extracellular acidification rate

The oxygen consumption rate (OCR) and extracellular acidification rate (ECAR) were analyzed using an XF24 extracellular flux analyzer (Seahorse Biosciences) according to the manufacturer's instructions. Neuronal cells were incubated with assay culture medium (XF Base medium containing 2 mM l-glutamine). Mitochondrial oxygen consumption was measured by the addition of oligomycin (1 μM, Seahorse Bioscience), carbonyl cyanide-p-trifluoromethoxyphenylhydrazone (FCCP) (1 μM), and fisetinone/antimycin A (0.5 μM) to assess maximal mitochondrial respiration. The culture medium was treated with glucose (10 mM), oligomycin (1 μM) and 2-deoxyglucose (50 mM, Sigma Aldrich) to measure the rate of extracellular acidification to assess glycolytic capacity.

### ChIP-seq

ChIP assays were performed as previously mentioned. Cells were fixed with 1% formaldehyde and lysed by sonication with 500 μL of cell lysate. The supernatant was delicately transferred to a new EP tube after centrifugation at 12,000 rpm for 10 min H3K18la antibody was added to the supernatant and incubated at 4 °C overnight. Activated magnetic beads were added, and the mixture was incubated at 4 °C for 1 h. The mixture was allowed to stand and the supernatant was removed. After elution and reverse cross-linking, DNA purification (A1701A, Takara) was performed to pull down the proteins and release the DNA from the bound chromatin. The target size fragments were recovered using magnetic beads and amplified by PCR to obtain the libraries to be sequenced; the constructed libraries were quality checked using agarose electrophoresis; the libraries were quantified using Qubit 2.0, and the different libraries were sequenced on the MGISEQ-T7 sequencer according to the effective concentration and the target amount of down-sequencing data needed.

### RNA sequencing and functional enrichment analysis

Total RNA was extracted from cultured cell samples using TRIzol reagent (Invitrogen, Carlsbad, CA) according to the manufacturer's protocol. We assessed RNA integrity using an Agilent 2200 TapeStation (Agilent Technologies, Santa Clara, CA). Subsequently, purified RNA was subjected to first- and second-strand cDNA synthesis, followed by ligation of adapters and low-cycling enrichment, according to the instructions for Illumina's NEBNext Ultra RNA Library Preparation Kit (NEB, Ipswich, MA). RNA concentrations were ascertained by means of a Qubit 2.0 fluorometer and a Qubit RNA assay kit (Life Technologies, Carlsbad, CA). Next, we used the Illumina TruSeq RNA Sample Preparation Kit (San Diego, CA) from 100 ng of high-quality RNA (OD260/280 = 1.8–2.2, OD260/230 ≥ 2.0, RIN ≥ 6.5, 28S:18S ≥ 1.0 and > 1 μg) to construct sequencing libraries and sequenced the libraries using the Illumina HiSeq 2500 platform (San Diego, CA). The mRNA levels of the unigenes were identified using TopHat v2.0.9 and Cufflinks software, and the exon model fragments per kilobase of exon model per million mapped reads (FPKM) were used for normalization to obtain clean reads after removing reads containing adapters, poly-N, and low quality from the raw data. Significantly differentially expressed genes between the control, LPS, and LPS + pc-Glis1 groups were assessed by DESeq. Differentially expressed genes (DEGs) were identified using the criteria of fold change greater than 2 and an adjusted *p* value of less than 0.05. Heatmap analysis and Kyoto Encyclopedia of Genes and Genomes (KEGG) pathway enrichment analysis were conducted for all DEGs.

### Molecular docking

Molecular docking simulations were performed to predict the binding mode between the unmodified H3K18-containing peptide and the catalytic domains of candidate histone acetyltransferases (p300, GCN5, MOF, and TIP60). The high-resolution 3D crystal structures of p300, GCN5, MOF, and TIP60 (PDB IDs: 5LKZ, 5TRM, 4DNC, 2OU2, respectively) were obtained from the RCSB Protein Data Bank (www.rcsb.org). The 2D structure of the target peptide was delineated using ChemBioDraw Ultra 14.0 and subsequently transformed into an energy-minimized 3D conformation using ChemBio3D Ultra 14.0 software. Molecular docking was executed using AutoDock Vina. The search grid for the p300 catalytic pocket was defined with center_x: 5.898, center_y: 34.097, and center_z:-13.542 and dimensions size_x: 82, size_y: 85, and size_z: 81. Correspondingly, the search grids for GCN5 (center_x: 32.804, center_y: 19.718, center_z: 45.185; size_x: 42, size_y: 43, size_z: 47), MOF (center_x: −5.476, center_y: 14.957, center_z: 17.174; size_x: 48, size_y: 77, size_z: 61), and TIP60 (center_x: 35.71, center_y: 17.08, center_z: 12.981; size_x: 52, size_y: 55, size_z: 77) were accurately assigned. Default parameters were applied for the docking runs. The best-scoring docking poses were selected, and the binding affinities were further evaluated using PRODIGY v2.0.

### Statistical analysis

Experimental data were analyzed using SPSS 22.0 (IBM, NY, USA) statistical software, and data were expressed as the mean ± SD. When the data obeyed a normal distribution (Shapiro–Wilk *W* test) and the variance was homogeneous, a *t* test was used to compare the data between two groups, and one (multi)factor analysis of variance (ANOVA) was used to compare the data between multiple groups. When the data did not obey a normal distribution or variance was not uniform, the Wilcoxon rank sum test was adopted to make comparisons of the data between two groups, and the Kruskal–Wallis test was employed for the comparison of the data among multiple groups. Tukey's test was used for multiple comparisons. The two-sided test level was *α* = 0.05. *P* values less than 0.05 were considered statistically significant. Figdraw and GraphPad Prism 9 (GraphPad, San Diego, CA, USA) were used to plot the data. All *in vitro* experiments were repeated at least three times independently (biological replicates, *n* = 3).

## Results

### Neuronal senescence occurs following SCI, with elevated levels of lactate and H3K18 lactylation

At the beginning of the experiments, to verify the purity of the primary spinal cord neuronal cultures and exclude potential microglia and astrocyte contamination, co-immunofluorescence staining was performed on cells in the control group. Co-immunofluorescence staining for NeuN, Iba-1 and GFAP revealed that the vast majority of cells exhibited strong NeuN-positive signals. In contrast, Iba-1-positive and GFAP-positive signals were barely detectable, indicating a negligible presence of microglia and astrocytes (Fig. S1B). Quantitative analysis demonstrated that the proportion of NeuN-positive neurons exceeded 90%, confirming that our culture protocol yields high-purity spinal cord neurons suitable for subsequent experiments (Fig. S1C). Paramos-de-Carvalho et al^13^ first demonstrated the induction of cellular senescence following SCI. To verify the emergence of a senescence phenotype in neurons following SCI, neuronal cells were treated with LPS *in vitro*, a widely established model to mimic SCI-induced neuroinflammation and neuronal damage, and WB was used to detect SASPs,^29^^,^^30^ including p53, p21, p16, γH2X, IL-6, CCL2, and CXCL2. LPS treatment resulted in a significant upregulation of these SASP markers (Fig. 1A and B). Immunofluorescence analysis for SA-β-galactosidase, a senescence marker, corroborated the WB results, showing an increase in senescence features following SCI (Fig. 1C and D). To rigorously confirm that the observed senescence phenotype specifically occurs in neurons and to validate the basis of their susceptibility to LPS, we performed immunofluorescence co-staining experiments. As shown in Figure S1D and S1E, double staining for the neuronal marker NeuN and the senescence marker p21 revealed that p21 expression was significantly upregulated specifically within NeuN-positive cells in the LPS-treated group compared to controls. This confirms that senescence occurs in neuronal cells. Furthermore, since LPS is known to signal through TLR4,^31^ we examined its expression in our culture system. We observed a clear co-localization of TLR4 with NeuN (Fig. S1F and S1G), indicating that these neurons express the necessary receptor to respond directly to LPS stimulation, thereby facilitating the subsequent senescence process. Additionally, LPS treatment promoted neuronal viability (Fig. 1E). To further substantiate senescence following SCI, spinal cord tissues from the Sham and SCI groups were analyzed using WB to detect SASP expression, confirming the *in vitro* findings of cellular senescence post-SCI (Fig. 1F and G). Immunofluorescence analysis of spinal cord tissues revealed more severe injury and elevated expression of senescence markers in the SCI group (Fig. 1H and I). Subsequent analysis of lactate levels showed a significant increase following LPS treatment (Fig. 1J), consistent with lactate's role as a precursor for lactylation.^8^ To investigate lactylation levels in neurons, WB and immunofluorescence were employed, revealing a substantial elevation in pan-lysine lactylation (Pan-Kla) levels following SCI (Fig. 1K–M). To further determine the cellular localization of the observed lactylation changes, we performed co-immunofluorescence staining using NeuN in combination with Pan-Kla and H3K18la. As shown in Figure S1H and S1I, the elevated signals of both Pan-Kla and H3K18la following LPS treatment strictly colocalized with NeuN-positive cells. These results confirm that the LPS-induced upregulation of global lactylation and H3K18la occurs specifically within spinal neurons. Histone lactylation at specific sites can regulate distinct pathophysiological processes.^32^ WB analysis using antibodies against various histone lactylation types was performed. The results indicated a significant increase in lactylation at multiple histone sites, with H3K18la showing the most prominent upregulation in neurons (Fig. 1N and O). Immunoprecipitation further identified H3K18la as the primary target for global histone lactylation (Fig. 1P). *In vivo* experiments using WB and immunofluorescence on spinal cord tissues confirmed the upregulation of Pan-Kla and H3K18la following SCI (Fig. 1Q–T). Next, we detected changes in lactylation after SCI using western blotting. The results showed an increase in Pan-Kla and H3K18la levels at 3, 7, 14, and 28 dpi (Fig. S3A and S3B). Studies have shown that neurons play a key role in SCI pathobiology and recovery.^9^ To further clarify whether neurons undergo lactylation after SCI, co-localization of NeuN with Pan-Kla was performed by immunofluorescence. Compared to the Sham group, the Pan-Kla signal intensity was considerably increased in neurons from 3 to 28 dpi (Fig. S3C and S3D). To further verify our hypothesis, we also used Glis1 conditional knockout (cKO) mice to conduct *in vivo* experiments (Methods in Supplementary Materials). Glis1^Nestin^-cKO mice were generated by crossing Glis1^f/f^ mice with Nestin-Cre transgenic mice (Fig. S4A–S4C). Indeed, as shown in Figure S4D and S4E, the expression of Glis1 was dramatically decreased in the spinal cords and other brain regions, such as the cortex, hippocampus and cerebellum, of Glis1 ^Nestin^-cKO mice compared with control mice. WB (Fig. S4F) and immunofluorescence results confirmed that Glis1 knockout significantly increased the senescence indicators and the levels of lactic acid in neurons (Fig. S4I–S4J). These results revealed that Pan-Kla and H3K18la were upregulated in the entire spinal cord and neurons after SCI. Overall, these results indicate that SCI promotes neuronal senescence, elevates lactate levels, and enhances H3K18 lactylation.

**Figure 1:**
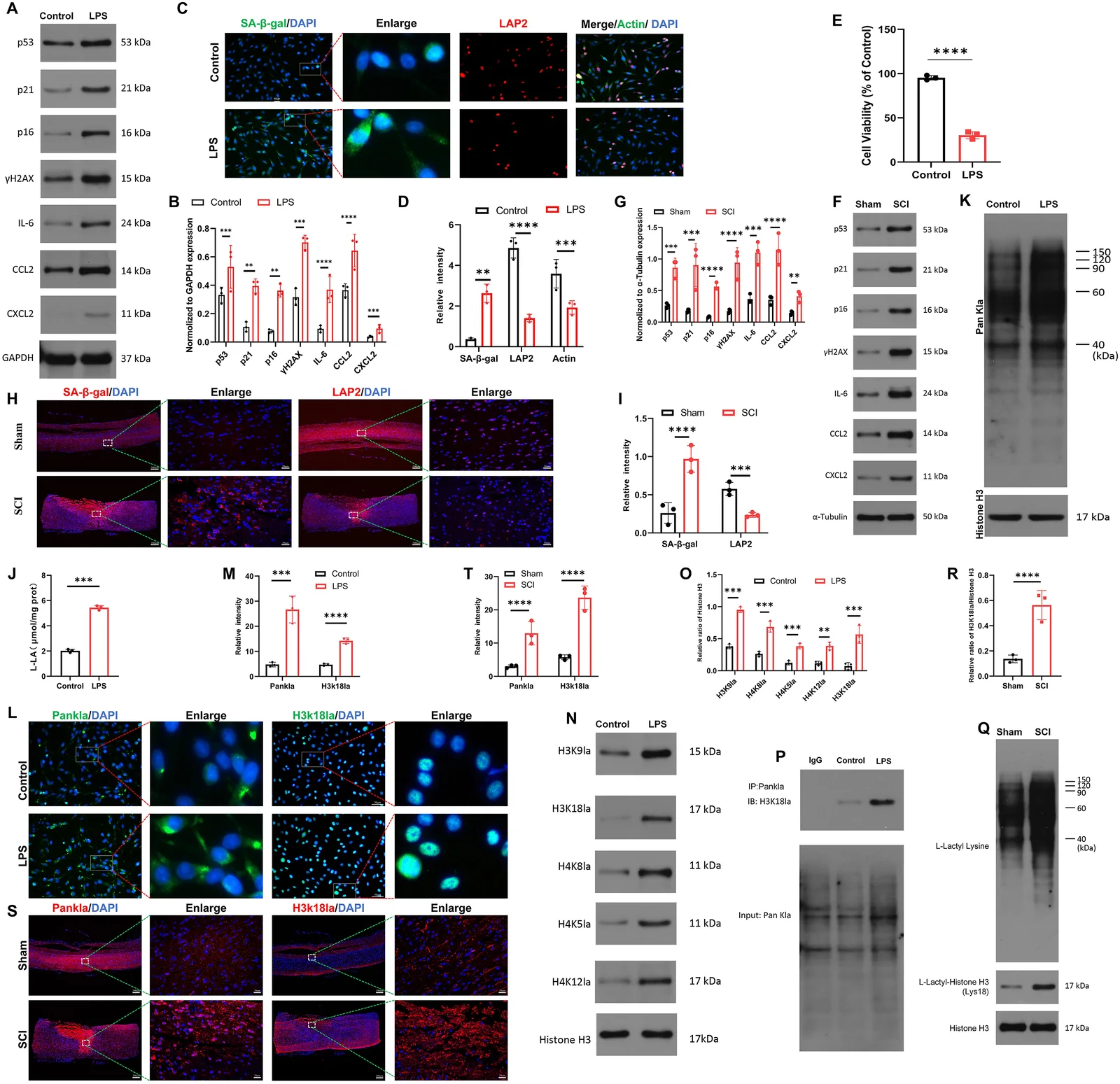
Neuronal senescence exists following SCI, with elevated levels of lactate and H3K18 lactylation. **(A, B)** After lipopolysaccharide (LPS) treatment, SASP expression levels and quantification results were detected by Western blot. **(C, D)** Immunofluorescence was used to detect the expression level and quantitative analysis of neuronal senescence markers. **(E)** Neuronal viability determined by CCK-8 assay in each group. **(F, G)** The expression level and quantitative analysis of SASP in the sham group and SCI group were detected by Western blotting (WB). **(H, I)** Representative images and quantification results of immunofluorescence detection of the expression levels of aging markers in spinal cord tissue. **(J)** Intracellular lactate content was measured using a lactate detection kit. **(K)** Global histone lactylation modification levels in neuronal cells measured by WB. **(L, M)** Representative images and quantification results of panlactylation and H3K18la levels of neuronal histones detected by immunofluorescence. **(N, O)** WB detection and quantitative analysis of histone lactylation modification sites in neuronal cells. **(P)** Co-immunoprecipitation of H3K18la. **(Q, R)** WB was used to detect and quantify the overall lactylation level and H3K18 lactylation level in the Sham group and the SCI group. **(S, T)** Representative images and quantitative results of histone lactylation before and after SCI detected by immunofluorescence. (*n* = 3. The relative contents were calculated using ImageJ software. All the data are expressed as the mean ± SD, and one- or two-way ANOVA followed by Tukey's post hoc test was applied. ∗*p* < 0.05, ∗∗*p* < 0.01, ∗∗∗*p* < 0.001, ∗∗∗∗*p* < 0.0001 *vs.* Control/Sham.

### Glis1 inhibits neuronal senescence by promoting H3K18 lactylation

Liu et al^14^ previously identified Glis1's role in regulating histone epigenetics, but its involvement in SCI, particularly regarding H3K18, remains unclear. To investigate Glis1 alterations following SCI, its expression was first analyzed using WB and immunofluorescence after LPS treatment, revealing a significant increase (Fig. 2A and B). We next assessed Glis1 expression at different time points after LPS treatment. PCR results confirmed the efficiency of plasmid-mediated overexpression and siRNA-mediated knockdown of Glis1 (Fig. S6A). WB results showed that the expression of Glis1 gradually decreased with increasing LPS treatment time (Fig. S5B), indicating that Glis1 may need to function in a specific time window, suggesting that such upregulation of endogenous Glis1 did not significantly reverse the senescence phenotype. The impact of Glis1 on neuronal senescence was then assessed by manipulating its expression through knockdown and overexpression. Additionally, Glis1 treatment restored neuronal viability, which indicated that Glis1 inhibits cell senescence, as evidenced by increased cell viability (Fig. 2C). Furthermore, WB and immunofluorescence analyses demonstrated that the knockdown of Glis1 significantly upregulated senescence markers (p53, p21, p16), while overexpression reversed these effects (Fig. 2D–F, H). Subsequent assessment of histone lactylation levels showed that Glis1 knockdown reduced both global and H3K18 lactylation induced by LPS, whereas overexpression markedly enhanced lactylation levels (Fig. 2G–J). Given the established link between glycolysis and histone lactylation,^33^ the regulatory influence of Glis1 on glycolytic metabolism was explored by measuring the expression of glycolysis-related proteins (c-MYC, LDHA, and GLUT1) in neurons with Glis1 knockdown or overexpression (Fig. 2K and L). The NAD^+^/NADH ratio (Fig. 2M), ECAR, and OCR (Fig. 2N–Q) were also evaluated. The results showed that Glis1 knockdown led to decreased expression of glycolysis-related proteins and a lower NAD^+^/NADH ratio, indicating a reduction in glycolytic flux. Conversely, Glis1 overexpression enhanced glycolysis, as reflected by a higher basal ECAR and glycolytic reserve, suggesting an increased glycolytic capacity. OCR analysis revealed that Glis1 overexpression lowered basal oxidative phosphorylation and spare respiratory capacity, indicating a shift toward glycolytic metabolism over oxidative pathways in neurons. These results imply that Glis1 influences neuronal metabolic reprogramming by increasing glycolytic reliance post-SCI, thereby regulating H3K18 lactylation. Collectively, these data suggest that in an LPS-induced inflammatory environment *in vitro*, Glis1 inhibits neuronal senescence through the promotion of glycolysis and subsequent modulation of H3K18 lactylation.

**Figure 2:**
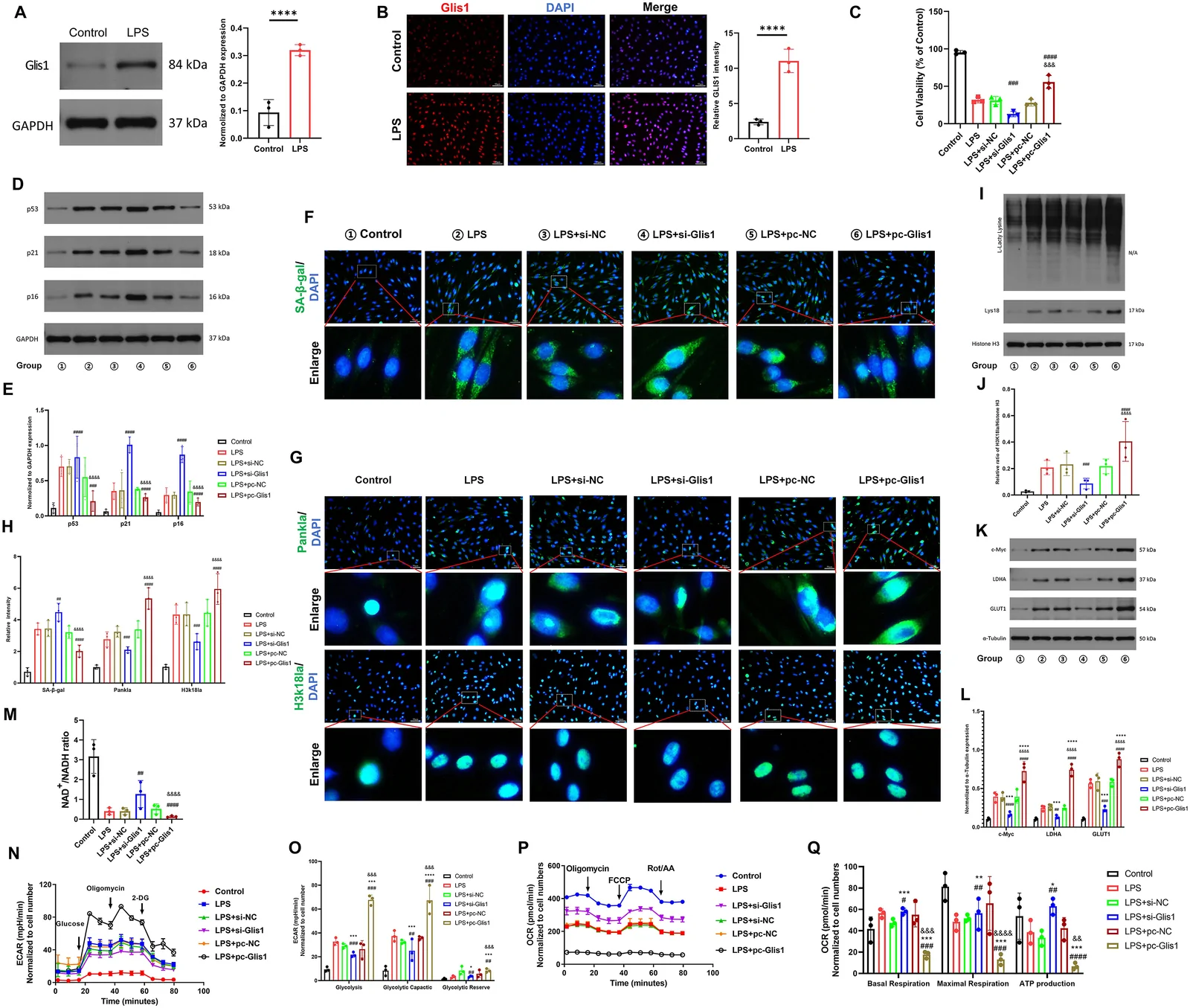
Glis1 inhibits neuronal senescence by promoting H3K18 lactylation. **(A)** After lipopolysaccharide (LPS) treatment, Glis1 expression levels and quantification results were detected by Western blot. **(B)** Immunofluorescence detection and quantification of Glis1 expression levels before and after LPS treatment. **(C)** Neuronal viability determined by CCK-8 assay in each group after treatment. **(D, E)** The expression levels and quantitative analysis of senescence markers of Glis1 after different treatments were detected by WB. **(F)** Representative images of the expression levels of senescence markers detected by immunofluorescence. **(G)** Representative images of neuron panlactylation and H3K18 lactylation levels detected by immunofluorescence. **(H)** Quantitative analysis results of representative immunofluorescence images. **(I, J)** Panlactylation and H3K18la levels were detected and quantified by WB. **(K, L)** Expression and quantitative analysis of glycolysis-related genes in neuronal cells after different treatments by WB. **(M)** Determination of the NAD^+^/NADH ratio in neuronal cells. **(N**–**Q)** Extracellular acidification rate (ECAR) and cellular oxygen consumption (OCR) of neuronal cells. (*n* = 3. The relative contents were calculated using ImageJ software. All the data are expressed as the mean ± SD, and one- or two-way ANOVA followed by Tukey's post hoc test was applied. ∗*p* < 0.05, ∗∗*p* < 0.01, ∗∗∗*p* < 0.001, ∗∗∗∗*p* < 0.0001 *vs.* Control; ^#^*p* < 0.05, ^##^*p* < 0.01, ^###^*p* < 0.001, ^####^*p* < 0.0001 *vs.* LPS; ^&^*p* < 0.05, ^&&^*p* < 0.01, ^&&&^*p* < 0.001, ^&&&&^*p* < 0.0001 *vs.* LPS + si-Glis1.

### SCG2 was identified as a potential downstream target of H3K18la, and H3K18la activated SCG2 transcription in neuronal cells

To elucidate the regulatory mechanism of H3K18 lactylation in gene expression, chromatin immunoprecipitation sequencing (ChIP-seq) was conducted on neuronal cells using an anti-H3K18la antibody. The ChIP-seq results revealed H3K18la enrichment in promoter regions, with multiple sequencing reads mapping to these areas (Fig. 3A–C). Pathway analysis using the Kyoto Encyclopedia of Genes and Genomes (KEGG) indicated that the H3K18la-specific genes were significantly enriched in pathways associated with cellular senescence (Fig. 3D), suggesting a role for histone lactylation in the regulation of senescence processes. To identify downstream targets coregulated by H3K18la and the upstream regulator Glis1 in neuronal cells following SCI, transcriptomic differences among the control, LPS, and LPS + pc-Glis1 groups were analyzed via RNA sequencing (RNA-seq). This involved total RNA extraction, RNA-seq transcriptome library construction, and identification of DEGs (Fig. S2A). A heatmap of the screened genes is shown in Fig. 3E. DEGs were selected based on a fold change > 2 and an adjusted *p* value < 0.05, followed by functional enrichment analysis using Gene Ontology (GO) and KEGG pathways (Fig. 3F and G). Gene set enrichment analysis (GSEA) indicated significant upregulation in angiogenesis, glycolysis, and pericyte growth signaling pathways, alongside downregulation of cell senescence pathways (Fig. S2B). Integrating ChIP-seq data with transcriptomic analysis enabled the identification of anti-H3K18la-ChIP target genes. These genes exhibited moderate upregulation after LPS treatment and a marked increase in expression following Glis1 overexpression, mirroring the expression pattern of Glis1 (Fig. 3H). Among the identified target genes, Secretogranin II (SCG2) was of particular interest due to its reported role in promoting angiogenesis following injury and its regulatory functions in aged neurons.^34^^,^^35^ To confirm its expression pattern, PCR analysis was performed across different experimental groups, revealing consistency with the differential gene expression observed in the RNA-seq data (Fig. S2C). The promoter region of SCG2 was then divided into three segments: region 1 (−1800 bp/−1200 bp), region 2 (−1200 bp/−600 bp), and region 3 (−600 bp/0 bp) (Fig. 3I). ChIP analysis was conducted on neuronal cells with and without Glis1 overexpression (pc-Glis1). Electrophoresis and PCR results indicated that H3K18la's positive regulatory binding site was located in region 1 (Fig. 3J and K). This finding was further validated by dual-luciferase reporter assays (Fig. 3L). Given previous evidence that glycolysis inhibitors can reduce histone lactylation levels^33^ (Fig. 3M), the glycolysis inhibitor OXAMATE was used to explore the regulatory effects of H3K18la on SCG2. The results showed that while pc-Glis1 enhanced dual-luciferase activity, OXAMATE treatment suppressed it (Fig. 3N). Additionally, ChIP analysis demonstrated that H3K18la enrichment in the SCG2 promoter region was significantly reduced by OXAMATE (Fig. 3O and P). To verify OXAMATE's inhibitory effects on glycolysis and lactate production, lactate levels in neuronal cells were measured following LPS and OXAMATE treatment, confirming a decrease in lactate with OXAMATE (Fig. 3Q). Subsequent WB analysis indicated that LPS treatment increased the expression of H3K18la and SCG2, while OXAMATE reversed this trend (Fig. 3R). We performed ChIP analysis of Glis1 and SCG2. The results showed that Glis1 was not enriched in the promoter region of SCG2 (Fig. S5C), and the regulation of Glis1 on SCG2 was indirect rather than direct. The PCR results supported these observations, demonstrating consistent regulatory changes (Fig. S2D). These results collectively indicate that SCG2 is a downstream target of the Glis1/H3K18la axis following SCI and is regulated through epigenetic modifications involving H3K18la.

**Figure 3:**
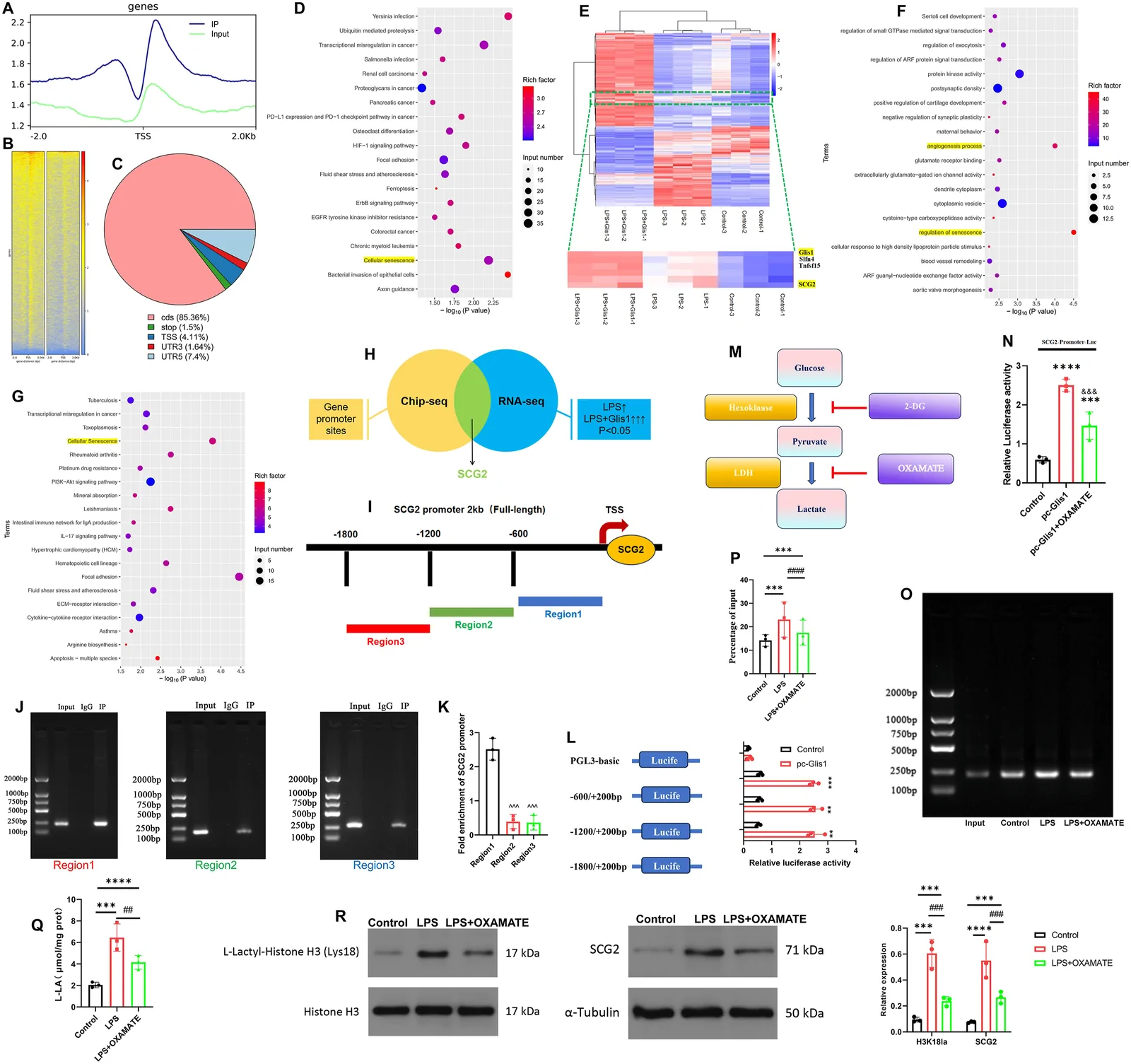
SCG2 was identified as a potential downstream target of H3K18la, and H3K18la activated SCG2 transcription in neuronal cells. **(A)** Distribution of H3K18la sites relative to the translation start site (TSS). **(B, C)** Distribution of peaks enriched by H3K18la on the genome of neuronal cells. **(D)** Analysis of the H3K18la peaks by the Kyoto Encyclopedia of Genes and Genomes (KEGG). **(E)** Heatmap of differentially expressed genes in the control group, the lipopolysaccharide (LPS) group, and the LPS + Glis1 group. **(F, G)** GO and KEGG enrichment analysis of the regulatory pathways in the LPS group and the LPS + pc-Glis1 group. **(H)** The process of screening SCG2 as a potential downstream target regulated by H3K18la through bioinformatics analysis. **(I)** Schematic diagram of the division of the SCG2 promoter region. **(J, K)** ChIP analysis results between H3K18la and each region of the SCG2 promoter. **(L)** A series of plasmids containing the 5′ unidirectional deletion of the SCG2 gene promoter region fused with the luciferase gene were transfected into neuronal cells. **(M)** Schematic diagram of the inhibition of histone lactylation by glycolysis inhibitors. **(N)** Double luciferase assays for H3K18la and SCG2 with or without OXAMATE and pc-Glis1 treatment. **(O, P)** ChIP analysis of H3K18la and SCG2 with or without OXAMATE treatment. **(Q)** Detection of intracellular lactate content with or without OXAMATE treatment using a lactate detection kit. **(R)**Western blotting detection of the expression levels and quantification of H3K18la and SCG2 with or without OXAMATE treatment (*n* = 3). All the data are expressed as the mean ± SD, and one- or two-way ANOVA followed by Tukey's post hoc test was applied. ∗*p* < 0.05, ∗∗*p* < 0.01, ∗∗∗*p* < 0.001, ∗∗∗∗*p* < 0.0001 *vs.* Control; ^#^*p* < 0.05, ^##^*p* < 0.01, ^###^*p* < 0.001, ^####^*p* < 0.0001 *vs.* LPS; ^&^*p* < 0.05, ^&&^*p* < 0.01, ^&&&^*p* < 0.001, ^&&&&^*p* < 0.0001 *vs.* pc-Glis1; ^ˆ^*p* < 0.05, ^^^*p* < 0.01,^^^^*p* < 0.001, ^^^^^*p* < 0.0001 *vs.* Region1.

### SCG2 is capable of undergoing metabolic reprogramming and forming a negative feedback loop with H3K18la, thereby regulating neuronal senescence

To elucidate the regulatory mechanism of SCG2 on neuronal aging and its potential effects on upstream factors such as H3K18la, Glis1, or glycolytic metabolism, SCG2 was overexpressed, and ECAR and OCR experiments were conducted. The results showed that in the LPS + pc-SCG2 group, ECAR levels were lower and OCR levels were higher than those in the LPS group (Fig. 4A–D). Additionally, lactate measurements indicated a significant decrease in lactate content in the LPS + pc-SCG2 group relative to the LPS group (Fig. 4E). These results suggest that SCG2 upregulation may promote metabolic reprogramming by reducing glycolysis, decreasing lactate production, and enhancing aerobic respiration. To assess the impact of SCG2-induced metabolic changes on H3K18 lactylation, ChIP analysis was performed to evaluate H3K18la binding at the SCG2 promoter in cells with SCG2 overexpression, with and without lactate treatment. The results indicated that SCG2 overexpression led to reduced enrichment of H3K18la in the SCG2 promoter region, a change that was reversed by lactate addition (Fig. 4F). This suggests that increased SCG2 expression inhibits H3K18la-mediated transcriptional activation, establishing a negative feedback regulatory mechanism. WB analysis was performed to measure the expression levels of Glis1, H3K18la, and glycolysis-related proteins. The results showed that while Glis1 levels remained unchanged compared to those in the LPS group, there was a significant decrease in the levels of H3K18la and glycolysis-related proteins after SCG2 overexpression (Fig. 4G–I). This implies that SCG2 regulates glycolytic metabolic reprogramming and forms a negative feedback loop with glycolysis and H3K18la, rather than directly affecting Glis1. To determine the role of SCG2 in neuronal aging, WB and immunofluorescence analyses were conducted to detect aging markers, revealing that increased SCG2 expression inhibited the aging phenotype (Fig. 4H–K). Then, to further confirm the negative feedback regulation of SCG2, we constructed SCG2 overexpression vectors with exogenous tags and performed exogenous SCG2 overexpression. PCR and WB were used to verify the changes in the expression level of endogenous SCG2 after overexpression of exogenous SCG2. The results showed that exogenous SCG2 inhibited the increase in endogenous SCG2 caused by Glis1 overexpression. The proposed negative feedback regulatory mechanism of SCG2 was confirmed (Fig. S5D). These results suggest that in an LPS-induced inflammatory environment *in vitro*, SCG2 expression mitigates neuronal aging and establishes a negative feedback mechanism involving glycolytic metabolism and H3K18 lactylation.

**Figure 4:**
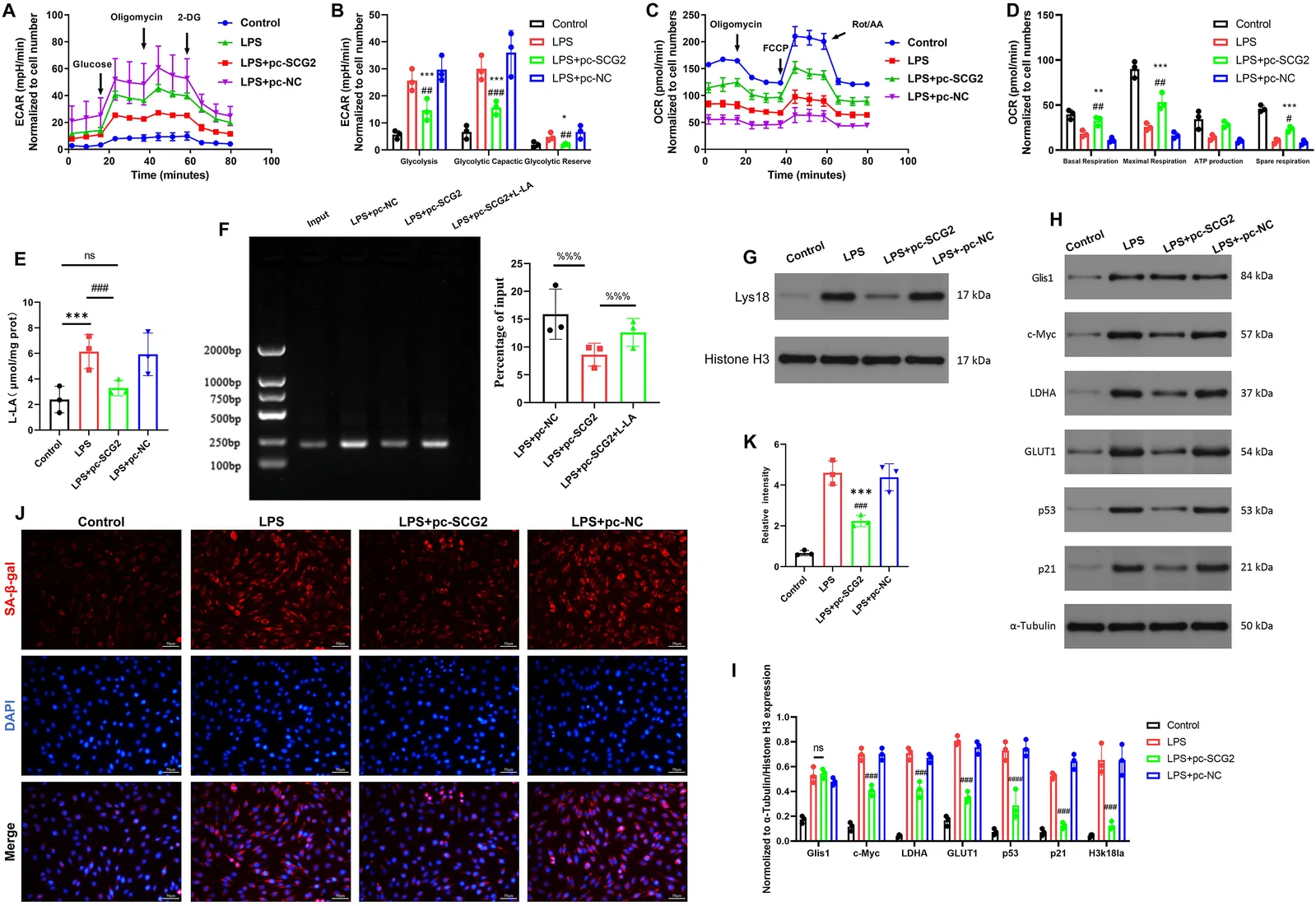
SCG2 is capable of undergoing metabolic reprogramming and forming a negative feedback loop with H3K18la, thereby regulating neuronal senescence. **(A**–**D)** Extracellular acidification rate (ECAR) and oxygen consumption rate (OCR) experiments of neuronal cells treated with or without lipopolysaccharide (LPS) and pc-SCG2. **(E)** Detection of intracellular lactate content in cells treated with or without LPS and pc-SCG2 using a lactate detection kit. **(F)** Chromatin immunoprecipitation analysis of the binding of the SCG2 promoter to H3K18la after overexpression of SCG2 and lactate treatment. **(G**–**I)** Western blotting detection and quantitative analysis of the contents of Glis1, H3K18la, aging markers, and glycolysis-related proteins. **(J, K)** Representative images and quantitative results of SA-β-gal levels analyzed by immunofluorescence (*n* = 3). The relative contents were calculated using ImageJ software. All the data are expressed as the mean ± SD, and one- or two-way ANOVA followed by Tukey's post hoc test was applied. ∗*p* < 0.05, ∗∗*p* < 0.01, ∗∗∗*p* < 0.001, ∗∗∗∗*p* < 0.0001 *vs.* Control; ^#^*p* < 0.05, ^##^*p* < 0.01, ^###^*p* < 0.001, ^####^*p* < 0.0001 *vs.* LPS; ^%^*p* < 0.05, ^%%^*p* < 0.01, ^%%%^*p* < 0.001, ^%%%%^*p* < 0.0001 *vs.* LPS + pc-SCG2.

### Glis1 can modulate p300, thereby promoting H3K18 lactylation

Previous studies have identified lactylation as a novel epigenetic modification, with histone acetyltransferase (HAT) enzymes, also referred to as writers, potentially playing a role in this process, similar to histone lysine acetylation.^36^ However, the specific HATs that promote histone lactylation in neuronal cells post-SCI remain unidentified. Four HAT family members—p300, MOF, TIP60, and GCN5—previously studied in lactylation research, were selected to investigate their potential roles in histone lactylation regulation in neuronal cells following SCI. Molecular docking experiments were first conducted to predict the binding patterns between the unmodified H3K18-containing peptide and the catalytic pocket of each candidate HAT (Fig. 5A). WB revealed that p300 exhibited the most significant change in expression after LPS treatment (Fig. 5B and C). Given p300's established role as a key enzyme in promoting histone lactylation and acetylation,^37^ it was hypothesized that p300 mediates lactylation following SCI. Co-immunoprecipitation experiments confirmed the interaction between p300 and H3K18la (Fig. 5D), and immunofluorescence colocalization further validated their spatial association (Fig. 5E). To explore the regulatory effects of Glis1 and p300 on H3K18 lactylation and downstream SCG2 expression, overexpression experiments were conducted. Immunofluorescence results indicated increased levels of p300 and H3K18la following LPS treatment; overexpression of Glis1 led to further upregulation of p300, H3K18la, and SCG2, while Glis1 expression remained stable (Fig. 5F and G). These results suggest that Glis1 influences downstream H3K18la and SCG2 expression via p300. Measurements of lactate levels supported the role of Glis1 and p300 in enhancing H3K18 lactylation (Fig. 5H). Given p300's dual role in facilitating both histone lactylation and acetylation, WB analysis was performed to assess histone acetylation levels. Although H3K18ac and H3K27ac levels increased with LPS treatment and Glis1/p300 overexpression, the changes were less pronounced compared to H3K18la (Fig. 5I). Co-immunoprecipitation confirmed that the binding of H3K18ac and H3K27ac to p300 was largely unaffected by LPS treatment or Glis1 overexpression, whereas the binding of H3K18la to p300 significantly increased under these conditions (Fig. 5J). These results demonstrate that in neuronal cells subjected to LPS treatment, Glis1 promotes H3K18 lactylation via p300 rather than acetylation. In summary, p300 interacts with H3K18la, and Glis1 regulates p300 to enhance the lactylation of H3K18 and the expression of downstream SCG2.

**Figure 5:**
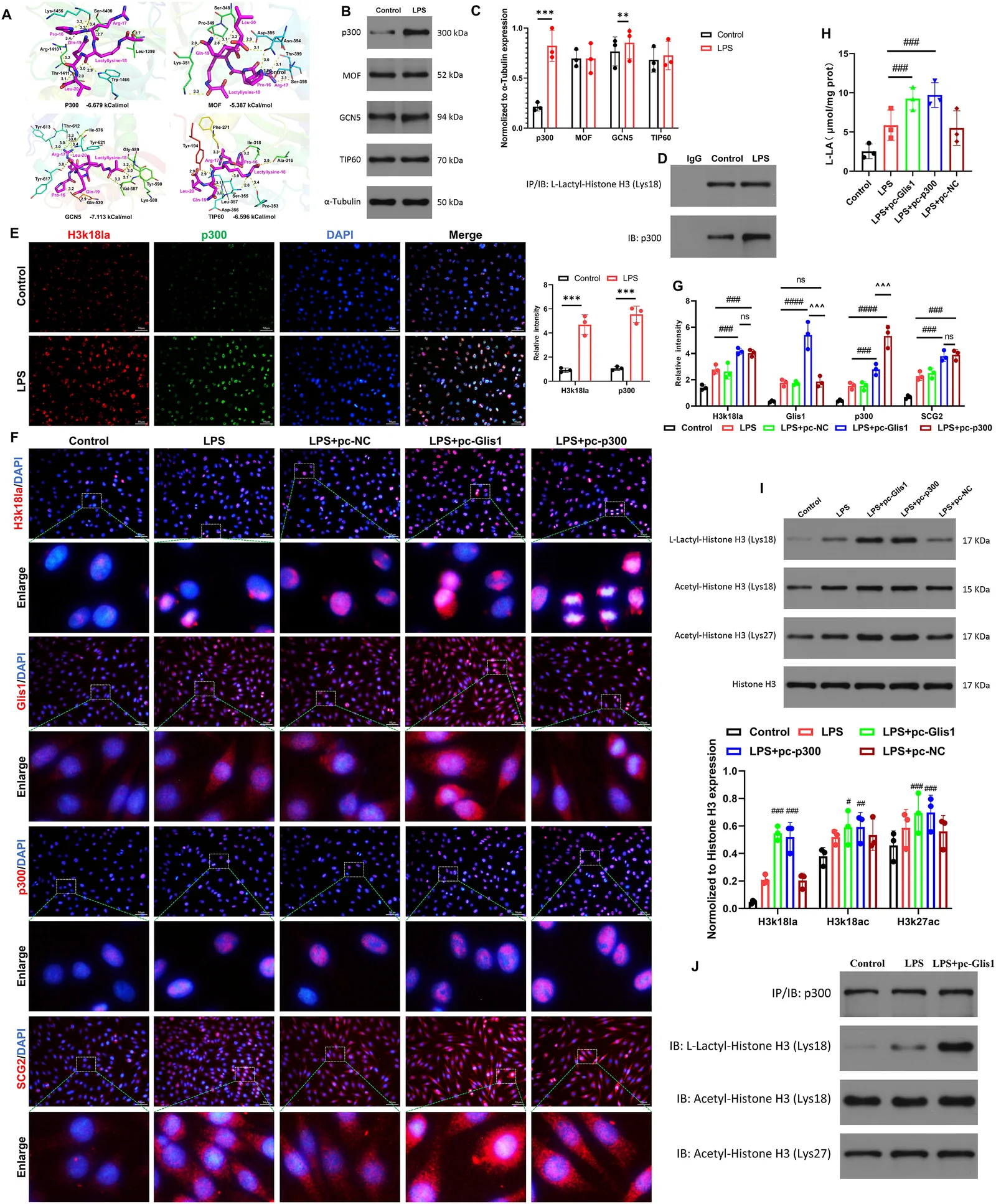
Glis1 can modulate p300, thereby promoting H3K18la lactylation. **(A)** The potential binding modes and affinities between the unmodified H3K18 peptide and candidate enzymes (p300, MOF, GCN5, and TIP60) were predicted using AutoDock Vina. **(B, C)** The expression levels of p300, MOF, GCN5, and TIP60 after lipopolysaccharide (LPS) treatment were detected by Western blotting and quantitatively analyzed. **(D)** The binding of H3K18la and p300 was verified by co-immunoprecipitation. **(E)** Representative images and quantitative analysis of the colocalization of H3K18la and p300 by immunofluorescence were performed. **(F, G)** Representative images and quantitative results of the levels of Glis1, p300, H3K18la, and SCG2 after overexpression of Glis1 and p300 were analyzed by immunofluorescence. **(H)** The intracellular lactate content in cells treated with LPS and overexpressing Glis1 and p300 was detected using a lactate detection kit. **(I)** The expression levels of H3K18la, H3K18ac, and H3K27ac after LPS and overexpression of Glis1 and p300 were detected by Western blotting and quantitatively analyzed. **(J)** The binding of H3K18la, H3K18ac, and H3K27ac with p300 after LPS and overexpression of Glis1 was verified by co-immunoprecipitation (*n* = 3). All the data are expressed as the mean ± SD, and one- or two-way ANOVA followed by Tukey's post hoc test was applied. ∗*p* < 0.05, ∗∗*p* < 0.01, ∗∗∗*p* < 0.001, ∗∗∗∗*p* < 0.0001 *vs.* Control; ^#^*p* < 0.05, ^##^*p* < 0.01, ^###^*p* < 0.001, ^####^*p* < 0.0001 *vs.* LPS; ^ˆ^*p* < 0.05, ^^^*p* < 0.01,^^^^*p* < 0.001, ^ˆ^^^^*p* < 0.0001 *vs.* LPS + pc-Glis1.

### Glis1 regulates the expression of SCG2 by promoting the expression of p300/H3K18la, thereby inhibiting aging and promoting angiogenesis

To verify the regulatory mechanism of Glis1/p300/H3K18la on neuronal aging in more detail, we overexpressed Glis1 and added the glycolysis inhibitor OXAMATE for verification. First, we employed Western blotting to detect p300, H3K18la, SCG2, and aging-related markers. The results revealed that the expression of p300, H3K18la, and SCG2 increased after overexpression of Glis1, which was consistent with previous verification results. However, the addition of OXAMATE reversed this phenomenon (Fig. 6A). Immunofluorescence also demonstrated similar results (Fig. 6B and C). Furthermore, cell viability assays demonstrated that while Glis1 preserved neuronal survival against LPS-induced senescence, pharmacological inhibition of glycolysis abolished this protective effect (Fig. 6H). Given the critical role of vascular regeneration in SCI repair^38^ and the known angiogenic potential of the Glis1 downstream target SCG2,^39^ we conducted cell migration and tube formation assays on HUVECs cultured in neuronal conditioned medium (CM) to evaluate the paracrine effects of Glis1. We found that CM derived from LPS-treated neurons impaired HUVEC migration, whereas CM from Glis1-overexpressing neurons promoted cell migration and counteracted the detrimental paracrine effects of LPS. However, pharmacological inhibition of glycolysis with OXAMATE in neurons abolished this promigratory effect (Fig. 6D and E). In addition, we found that wound healing assays of HUVECs cultured in CM derived from LPS-treated and SCG2-overexpressing neurons exhibited enhanced cell migration, further supporting the proangiogenic role of neuron-derived SCG2 in SCI recovery (Fig. S5E). Furthermore, tube formation assays revealed that HUVECs cultured in CM from Glis1-overexpressing neurons exhibited a significantly increased tube formation rate. Conversely, tube formation was markedly inhibited when using CM from neurons cotreated with the glycolysis inhibitor (Fig. 6F and G). These results suggest that the Glis1-mediated metabolic axis in neurons is essential for maintaining endothelial function via paracrine signaling. In conclusion, this part of the study indicates that in the LPS-induced inflammatory microenvironment *in vitro*, Glis1 can promote the expression of p300/H3K18la to regulate the expression of SCG2, thereby inhibiting aging and promoting angiogenesis.

**Figure 6:**
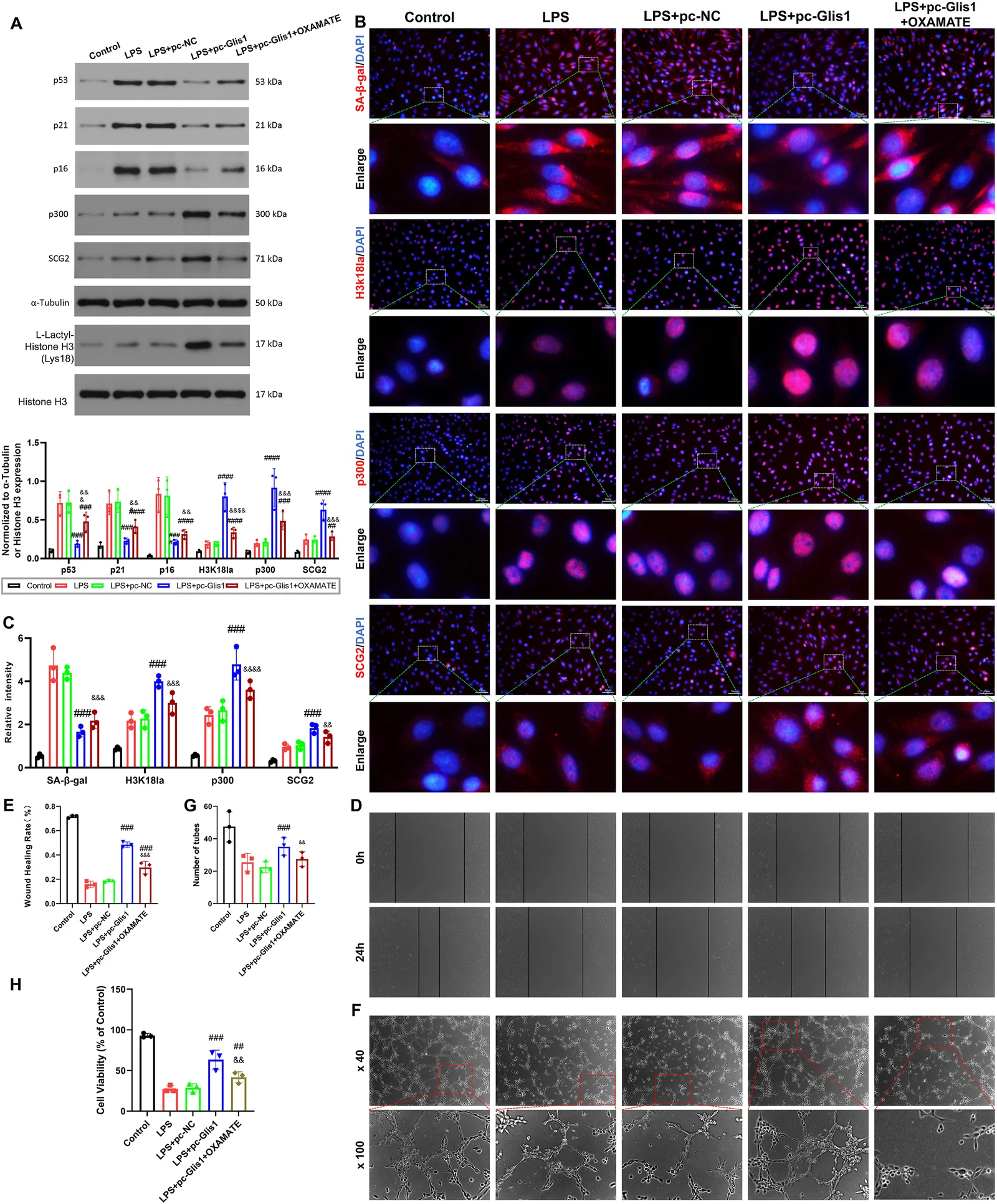
Glis1 regulates the expression of SCG2 by promoting the expression of p300/H3K18la, thereby inhibiting aging and promoting angiogenesis. **(A)** Western blotting was employed to detect the expression levels and quantitative results of p300, H3K18la, SCG2, and aging-related markers after lipopolysaccharide (LPS) treatment, overexpression of Glis1, and treatment with or without OXAMATE. **(B, C)** Immunofluorescence was utilized to detect representative images and quantitative analysis of the expression of p300, H3K18la, SCG2, and aging-related markers after LPS treatment, overexpression of Glis1, and with or without OXAMATE treatment. **(D, E)** Migration assays of HUVECs cultured in CM derived from primary neurons with the indicated treatments (control, LPS, LPS + pc-Glis1, and LPS + pc-Glis1+OXAMATE). **(F, G)** Tube formation assays of HUVECs cultured in neuronal CM from the respective treatment groups (scale bar: 200 μm). **(H)** Neuronal viability determined by CCK-8 assay in each group after LPS treatment, overexpression of Glis1, and OXAMATE treatment (*n* = 3). The relative contents were calculated using ImageJ software. All the data are expressed as the mean ± SD, and one- or two-way ANOVA followed by Tukey's post hoc test was applied. ∗*p* < 0.05, ∗∗*p* < 0.01, ∗∗∗*p* < 0.001, ∗∗∗∗*p* < 0.0001 *vs.* Control; ^#^*p* < 0.05, ^##^*p* < 0.01, ^###^*p* < 0.001, ^####^*p* < 0.0001 *vs.* LPS; ^&^*p* < 0.05, ^&&^*p* < 0.01, ^&&&^*p* < 0.001, ^&&&&^*p* < 0.0001 *vs.* LPS + pc-Glis1.

### Glis1 promotes the expression of p300/H3K18la in rats, thereby regulating the expression of SCG2, inhibiting aging, and alleviating SCI

The *in vivo* expression and regulatory role of the Glis1/p300/H3K18la pathway were further investigated using a rat SCI model. AAV vectors carrying shRNA targeting Glis1 were injected at the injury site. WB results confirmed that the knockdown efficiency of sh-Glis1 was feasible and effective (Fig. S5A). We performed co-immunofluorescence staining on spinal cord tissue sections. As shown in Figure S6C, we observed a significant colocalization of the neuronal marker NeuN (neuron-specific nuclear protein) with both H3K18la and p21. Quantitative observation confirmed that the majority of cells exhibiting high levels of lactylation and senescence signals were NeuN-positive. These data provide direct histological evidence that neurons are the specific cell type undergoing metabolic reprogramming and senescence following SCI, further supporting the physiological relevance of our *in vitro* model. T2-weighted MRI scans revealed more extensive spinal cord tissue injury in the SCI + sh-Glis1 group than in the SCI group, characterized by high signal intensity, uneven signal distribution, and indistinct boundaries (Fig. 7A). Quantitative analysis of MRI data yielded consistent results (Fig. S6B). WB was performed to assess the expression of p300, H3K18la, and SCG2 in the signaling pathway. The results indicated a significant reduction in H3K18 lactylation, p300, and SCG2 expression levels following Glis1 knockdown, consistent with the *in vitro* findings (Fig. 7B). Additionally, markers of cellular aging and neuronal regeneration were examined, showing that Glis1 knockdown promoted aging and diminished neuronal repair and regeneration (Fig. 7C and D). Immunofluorescence analysis corroborated these results. SA-β-gal staining, a senescence marker, was significantly increased in the SCI + sh-Glis1 group (Fig. 7E). The expression patterns of NF and GFAP indicated that NF-positive cells in the SCI + sh-Glis1 group were irregularly arranged and showed reduced density, while GFAP-positive cells were significantly increased, suggesting impaired axonal regeneration and increased glial fibrosis due to Glis1 knockdown (Fig. 7F and G). Tubulin beta-III (Tuj-1) staining showed a marked decrease in Tuj-1-positive cells in the SCI + sh-Glis1 group, with a sparse distribution at the injury site compared to the SCI and SCI + sh-NC groups, where these cells were more concentrated around the lesion (Fig. 7H). Regarding angiogenesis, CD31, an endothelial marker, showed significantly fewer positive cells in the SCI + sh-Glis1 group than in the other groups, with almost no tube-forming structures, indicating compromised angiogenesis (Fig. 7I). At 4 weeks post-SCI, spinal cord tissue sections were stained to assess histopathological changes. Hematoxylin-eosin (HE) staining showed that the inflammatory response and tissue damage were markedly elevated in the SCI + sh-Glis1 group compared to the other groups, with a significant increase in the number of inflammatory cells and pronounced tissue edema (Fig. 8A). Nissl staining revealed a substantial reduction in the number of Nissl bodies (indicated by red triangular arrows) in the SCI + sh-Glis1 group, with almost no detectable Nissl bodies, indicating severe neuronal damage (Fig. 8B). Masson staining highlighted extensive collagen fiber deposition at the SCI site, with the most severe fibrosis observed in the SCI + sh-Glis1 group (Fig. 8C). Gross examination of the spinal cord specimens showed the most severe damage in the SCI + sh-Glis1 group (Fig. 8D). The effect of Glis1 on motor function recovery following SCI was further evaluated using BBB scores. The SCI + sh-Glis1 group exhibited the poorest functional recovery, with only some rats able to support their body weight by the final assessment. In contrast, the SCI and SCI + sh-NC groups showed signs of improved motor function, such as ankle joint movement, and some rats could stand and walk on both hindlimbs, albeit with noticeable limping (Fig. 8E). Similarly, Glis1 cKO mice were used to verify the regulatory role of Glis1 after SCI. Consistent with the *in vivo* results of Glis1 knockdown by the AAV vector mentioned above, Glis1-cKO mice showed a significant reduction in SCG2 expression and a significant increase in glycolysis-related enzymes after SCI (Fig. S4F). NF and GFAP staining further indicated that Glis1 knockdown resulted in impaired axonal regeneration and enhanced neural fibrosis (Fig. S4G and S4H). Similar results as Figure 8 were obtained for HE and Nissl staining and BMS scores of mice (Fig. S4K–S4M). These results suggest that Glis1 knockdown significantly impairs H3K18 lactylation, as well as the expression of p300 and SCG2, thereby exacerbating cellular aging and SCI. Additionally, Glis1 knockdown delays motor function recovery and slows the repair process following SCI. Collectively, these results indicate that Glis1 plays a protective role in alleviating SCI *in vivo* by promoting H3K18 lactylation and facilitating the repair and recovery of spinal cord tissues.

**Figure 7:**
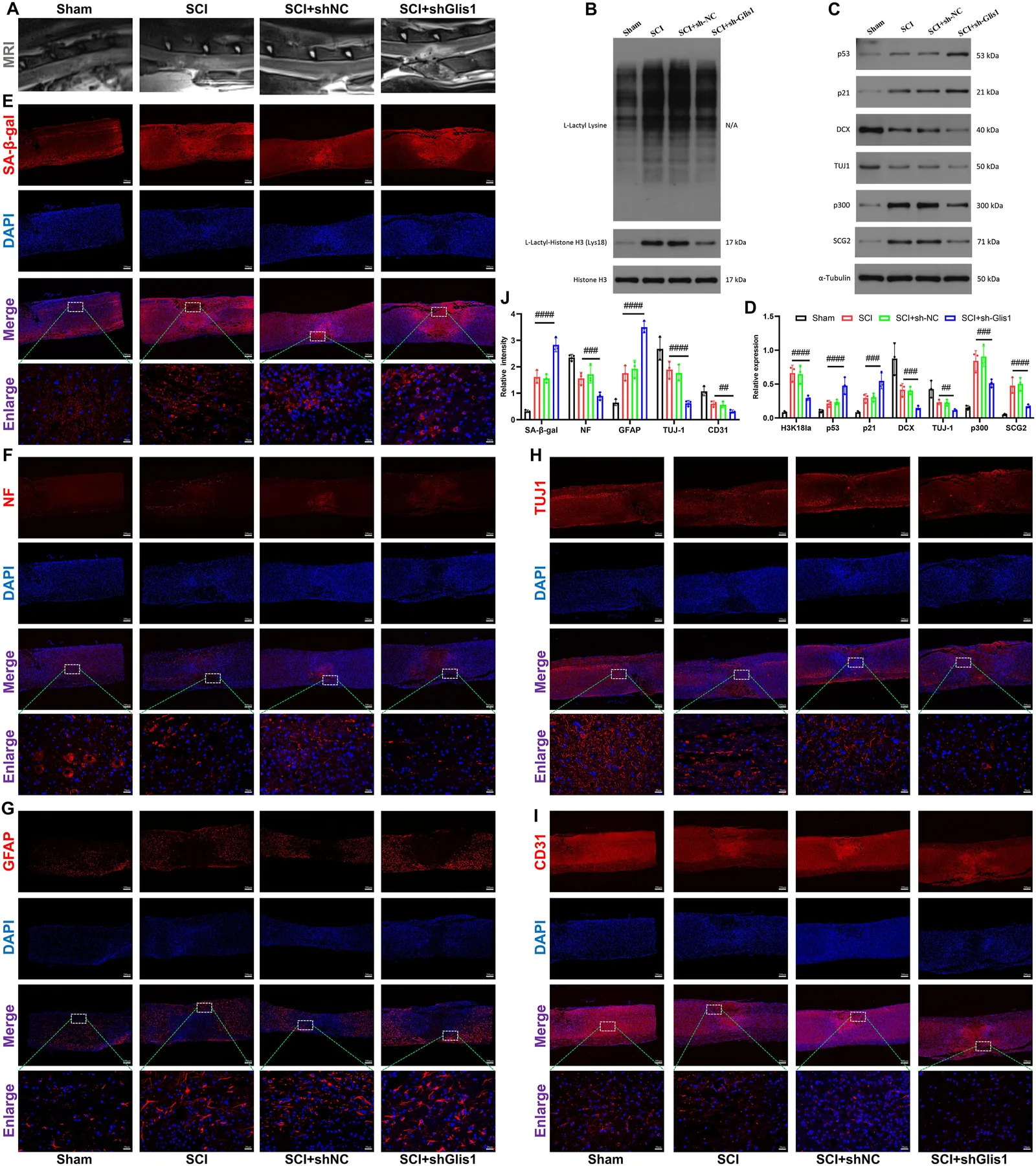
Glis1 promotes the expression of p300/H3K18la in rats, thereby regulating the expression of SCG2, inhibiting aging, and alleviating SCI. **(A)** Representative T2-weighted magnetic resonance (MR) images of the spinal cords in each group (*n* = 3). **(B, C)** Western blot detection of p300, H3K18la, SCG2, aging markers, and neuron regeneration markers in each group. **(D)** Quantitative analysis of p300, H3K18la, SCG2, aging markers, and neuron regeneration markers in spinal cord tissues after different treatments by WB. **(E)** Representative images of the expression level of SA-β-gal in spinal cord tissues detected by immunofluorescence. **(F)** Representative images of the expression level of NF in spinal cord tissues detected by immunofluorescence. **(G)** Representative images of the expression level of GFAP in spinal cord tissues detected by immunofluorescence. **(H)** Representative images of the expression level of Tuj-1 in spinal cord tissues detected by immunofluorescence. **(I)** Representative images of the expression level of CD31 in spinal cord tissues detected by immunofluorescence. **(J)** Quantitative analysis of immunofluorescence in each group. (The relative contents were calculated using ImageJ software. All the data are expressed as the mean ± SD, and one- or two-way ANOVA followed by Tukey's post hoc test was applied. ^#^*p* < 0.05, ^##^*p* < 0.01, ^###^*p* < 0.001, ^####^*p* < 0.0001 *vs.* Sham.

**Figure 8:**
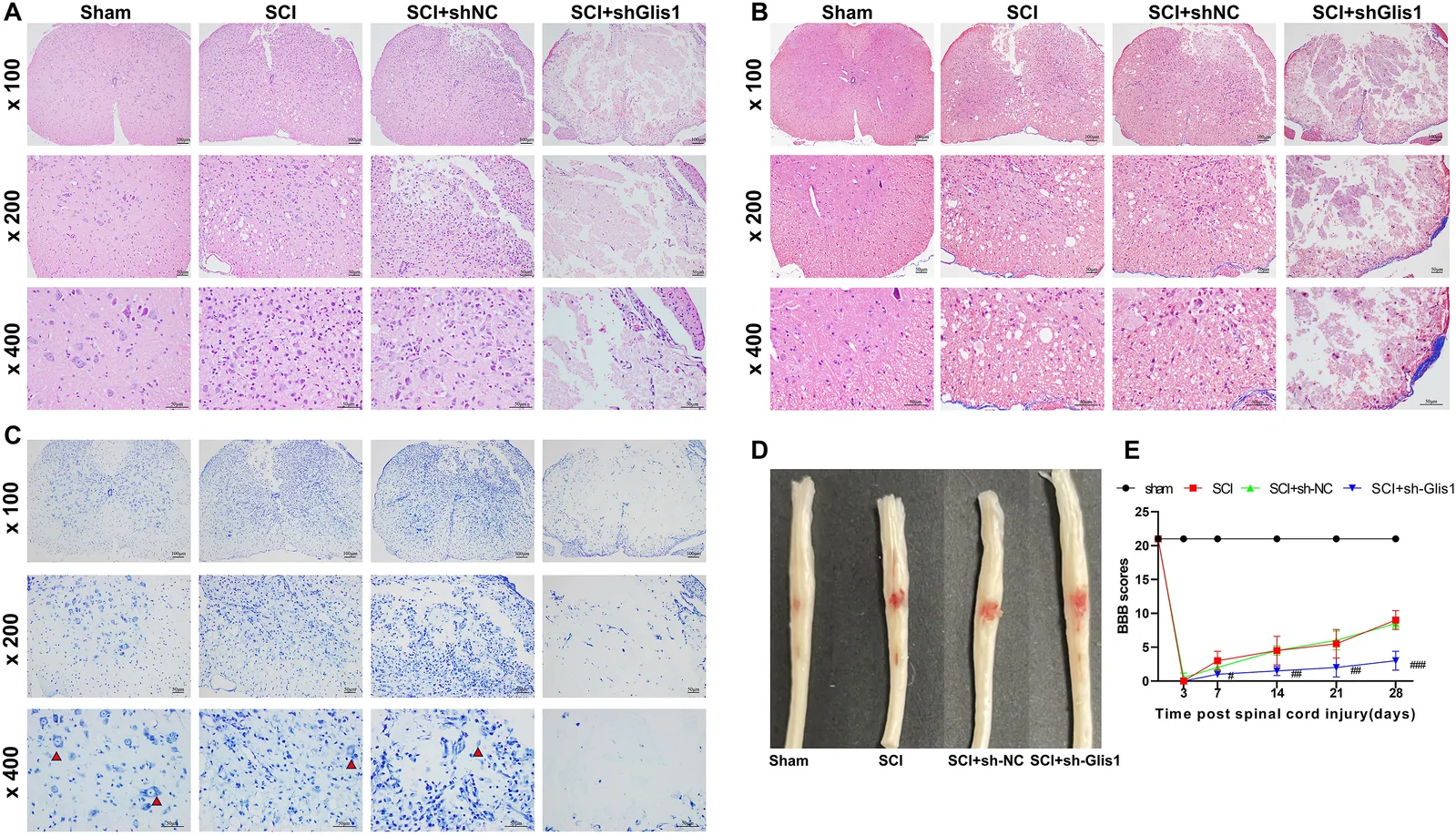
Specimen and pathological staining of the rat spinal cord. **(A**–**C)** Representative hematoxylin and eosin (H&E) (A), Nissl (B), and Masson staining (C) of spinal cord tissues in each group (*n* = 3). **(D)** The gross appearances of spinal cord tissues in each group. **(E)** BBB scores of different groups at 3, 7, 14, 21, and 28 days after SCI or sham surgery (*n* = 3). (The relative contents were calculated using ImageJ software. All the data are expressed as the mean ± SD, and one- or two-way ANOVA followed by Tukey's post hoc test was applied. ^#^*p* < 0.05, ^##^*p* < 0.01, ^###^*p* < 0.001, ^####^*p* < 0.0001 *vs.* Sham.

## Discussion

SCI is a devastating condition that often results in severe and lasting disability. The disruption of neuronal axons and subsequent neuronal death following SCI lead to dysfunction in neural circuits, with neural circuit plasticity being essential for functional recovery.^9^ Recent research has focused on neuron regeneration and repair strategies to improve long-term outcomes following SCI.^40^ It is well established that the aging response triggered by acute injury initiates tissue remodeling through the recruitment of immune cells via the SASP, indicating that SCI induces neuronal aging. Targeting the SASP to inhibit the aging process of these senescent cells has shown potential for promoting injury repair.^13^ For instance, Zou et al demonstrated that inhibiting ERCC6 could alleviate SCI by suppressing cellular senescence.^41^ Our study supports these results, revealing consistent results through *in vivo* and *in vitro* molecular biology experiments. The presence of SASP markers in neurons following SCI confirmed the occurrence of cellular senescence, suggesting that reducing neuronal aging may be a viable therapeutic target to lessen the injury response. Furthermore, it has been reported that SCI leads to the formation of a hypoxic microenvironment at the injury site, resulting in increased lactic acid production and metabolic shifts toward enhanced glycolysis.^5^ Lactate, as a substrate for histone lactylation, can induce this modification from both exogenous and endogenous sources, thereby activating downstream gene transcription.^42^ It was hypothesized that modulating lactate levels post-SCI could mitigate injury, and our results confirmed this hypothesis, showing increased lactate levels and a significant rise in global histone acetylation following SCI.

Gli-like transcription factor 1 (Glis1) exhibits reprogramming activity across various cell types and methods, indicating its significant clinical potential for modulating diverse cellular processes.^43^^,^^44^ The mechanism by which Glis1 regulates neuronal senescence following SCI warrants further investigation. Notably, Glis1 expression was found to increase in response to LPS treatment. To explore its function under *in vitro* conditions simulating the SCI environment, Glis1 was subjected to knockdown and overexpression manipulations. Consistent with previous studies, the results confirmed that Glis1 exerts an inhibitory effect on neuronal senescence. It is hypothesized that the damage induced by LPS triggers compensatory upregulation of Glis1, which, however, is insufficient to fully counteract the neuronal senescence caused by the injury. Histone lactylation has emerged as a key regulator in the pathophysiological processes of various diseases,^45^ although its role in SCI remains underexplored. Recently, Hu et al provided the first evidence that histone lactylation can ameliorate SCI,^46^ supporting findings from our own study. Histone lactylation relies on environmental lactate levels as a substrate for epigenetic modifications at specific histone residues. Among the histone marks, lactate-driven lysine lactylation has been shown to be particularly significant, with verified lactylation sites including H4K12, H3K18, H3K23, H4K5, and H4K8.^33^ Hu et al further identified a notable increase in H4K12la in microglia post-SCI, which influenced scar formation^46^. In the current study, overexpression of Glis1 was found to elevate global lactylation levels, with H3K18la showing the most prominent change among potential lactylation sites, a finding further validated by co-immunoprecipitation. This study provides the first evidence that Glis1 may regulate neuronal aging by promoting H3K18la, aligning with trends observed in previous research. However, our discovery that H3K18la serves as a key target for Glis1 in the regulation of neuronal aging following SCI contrasts with Hu's findings, suggesting that histone lactylation sites may be selectively regulated by different upstream molecules in various biological systems and that these modifications may differ according to cell type.

H3K18 is a critical site for lactylation modification, known for its role in regulating downstream gene transcription and influencing various disease processes.^46^ However, its function in neuronal cells following SCI has not been investigated. To address this gap, ChIP-seq and RNA-seq were conducted to identify the promoter regions bound by H3K18la, revealing SCG2 as a downstream target that follows a regulatory pattern consistent with upstream Glis1 expression. SCG2, a significant member of the chromogranin/secretogranin family, is rapidly processed into bioactive peptides within the tissues where it is expressed.^47^^,^^48^ It functions as an angiogenic cytokine capable of inhibiting endothelial apoptosis and promoting endothelial cell proliferation, migration, and angiogenesis.^34^^,^^49^ Emerging research highlights SCG2's essential role in neovascularization, wound healing, and tissue regeneration. Furthermore, SCG2 is increasingly recognized as a regulatory molecule in the nervous system.^50^ However, its regulatory effects following SCI have not been explored. ChIP and dual-luciferase assays confirmed the enrichment of H3K18la in the specified region of the SCG2 promoter. For further investigation, OXAMATE, a glycolysis inhibitor, was used to simulate the inhibition of lactylation. The results showed that LPS treatment and Glis1 overexpression increased H3K18la and SCG2 expression, as well as their interaction, whereas OXAMATE reversed these effects. These findings suggest that Glis1 regulates H3K18la, thereby activating the transcription of SCG2. Subsequent experiments confirmed that SCG2 exerts an inhibitory effect on neuronal aging.

Research indicates that the rate of lactylation is heavily influenced by the expression levels of lactylation-modifying enzymes^51^, and previous studies have shown the unique and important ability of p300 to utilize lactate as a lysine lactylation substrate^52^. This study examined key HAT family members, including p300, TIP60, MOF, and GCN5, for their roles in the regulation of H3K18la by Glis1. Glis1 itself leads to the activation of DNA sequence-specific transcription factors, initiating specific gene expression and signaling cascades.^14^^,^^53^ The findings demonstrated a significant catalytic effect of Glis1-dependent p300 recruitment on H3K18la and elucidated its potential role as an upstream regulatory element that regulates neuronal histone lactate and downstream senescence gene expression. p300 is significantly involved in promoting histone lactylation and activating target gene transcription. Additionally, Liu et al have shown that Glis1 can enhance histone acetylation,^14^ and HAT enzymes are traditionally known as acetylation modifiers.^33^ To clarify the type of epigenetic regulation mediated by Glis1 in neuronal cells, the levels of histone acetylation were assessed. Our results provide the first evidence that in the context of neuronal injury regulation, Glis1 primarily facilitates the lactylation of H3K18 via p300 rather than promoting acetylation.

Metabolic dysfunction is a key factor impeding recovery from SCI.^54^ Research has shown that metabolic remodeling of nerve cells can alleviate SCI, as cells reprogram their metabolism in response to the hypoxic environment postinjury, resulting in enhanced glycolysis and elevated lactate levels.^6^ Lactate, a metabolic byproduct, has been implicated in various biological functions^55^ and is increasingly recognized for its neuroprotective properties, contributing to the repair of neural tissue.^56^ Additionally, exogenous lactate administration has been found to improve neural function following central nervous system injuries.^57^ Previous studies suggest that cellular senescence is closely tied to oxygen availability, with higher oxygen concentrations accelerating the senescence process.^58^ Furthermore, metabolic shifts in senescent cells favor oxidative respiration over anaerobic glycolysis. Recently, it has been proposed that lactate derived from glycolytic metabolism can also drive cellular senescence.^59^ The relationship between cellular senescence and metabolism remains complex and somewhat ambiguous. This study aimed to elucidate how Glis1-mediated metabolic reprogramming regulates neuronal senescence following SCI. Our findings demonstrated that overexpression of Glis1 upregulated genes associated with glycolysis, consistent with its role in promoting H3K18 lactylation, echoing earlier observations by Liu et al.^14^ Further investigation revealed that overexpression of the downstream target SCG2 did not affect Glis1 expression but significantly inhibited the levels of glycolysis-related proteins and H3K18la. SCG2 overexpression also exerted a pronounced inhibitory effect on neuronal senescence. Measurements of ECAR and OCR indicated that SCG2 overexpression feedback-inhibited glycolysis while promoting cellular aerobic respiration. The data suggest that when cellular damage induces senescence, glycolysis and lactate modification levels increase. Glis1 enhances glycolysis, thereby regulating SCG2 expression and suppressing neuronal senescence. Once SCG2 expression reaches a critical threshold, it exerts a negative feedback effect, inhibiting glycolytic metabolism and lactate-induced H3K18 lactylation, thereby achieving metabolic reprogramming to restore metabolic equilibrium in neurons. These findings were supported by experimental data, representing an initial exploration and significant contribution to the field of metabolic epigenetics. To further validate the regulatory mechanism of the Glis1/p300/H3K18la/SCG2 pathway, OXAMATE, a glycolysis inhibitor, was employed to confirm the pathway's role in regulating H3K18 lactylation, inhibiting neuronal senescence, and promoting angiogenesis *in vitro*. Moreover, our findings extend the therapeutic potential of Glis1 beyond neuronal protection. The observation that Glis1 overexpression in neurons promotes endothelial cell migration and tube formation via paracrine signaling—an effect also abolished by neuronal glycolysis inhibition—suggests that the Glis1/H3K18la axis in neurons can profoundly influence the surrounding SCI microenvironment, including neighboring endothelial cells. By concurrently reprogramming the metabolism of both neurons and endothelial cells, Glis1 regulates a multifaceted repair process that encompasses both neuroprotection and revascularization. *In vivo* experiments further corroborated these findings, demonstrating that the pathway suppresses neuronal senescence, neuroinflammation, and fibrosis while promoting axonal regeneration and angiogenesis in rats following SCI.

There are some limitations in this study. First, Glis1, as a molecule promoting lactylation modification, may also regulate other lactylation modification “readers” and “erasers”, while this study only studied “writers” such as p300. Second, in the study of SCI, we only focused on the aging of neuronal cells, without considering other regulatory mechanisms, including those of microglia, astrocytes and glial immune crosstalk. We will further study these issues in the future. While ChIP-seq and RNA-seq *in vitro* models provide critical mechanistic insights, they cannot fully recapitulate the multicellular milieu and potentially omit microglial–neuronal interactions after SCI. Emerging technologies such as spatial transcriptomics (10x Visium HD) and single-nucleus RNA-seq of laser-captured neurons will be prioritized in future work to bridge this gap. Furthermore, as an *in vitro* cell model of SCI, LPS-treated neurons may not be able to fully simulate the changes in the local environment after SCI, and cross-validation of multiple models (such as the oxygen-glucose deprivation model) may be needed to improve the construction of *in vitro* SCI models. Finally, the animal experiment was validated by using AAV-mediated shRNA, but considering the uncertainty of shRNA, including low transfection efficiency, it may be more suitable to use gene knockout mice or transgenic mice.

In conclusion, this study demonstrates that Glis1 initiates H3K18 lactylation via p300 and regulates the expression of downstream SCG2. The negative feedback loop among the expression of SCG2, glycolytic metabolism, and H3K18 lactylation modification undergoes metabolic reprogramming, thereby suppressing neuronal senescence (Fig. 9). Our research results offer novel perspectives on the relationship among metabolism, senescence, and SCI and provide a new viewpoint for the repair strategies of neurons after SCI. These findings elucidate novel therapeutic targets within the pathological processes associated with SCI, and this pathway is crucial for neuroprotection and functional recovery postinjury. The results of this study provide promising and clinically translatable avenues for enhancing SCI therapies by targeting the amelioration of neuronal senescence.

**Figure 9:**
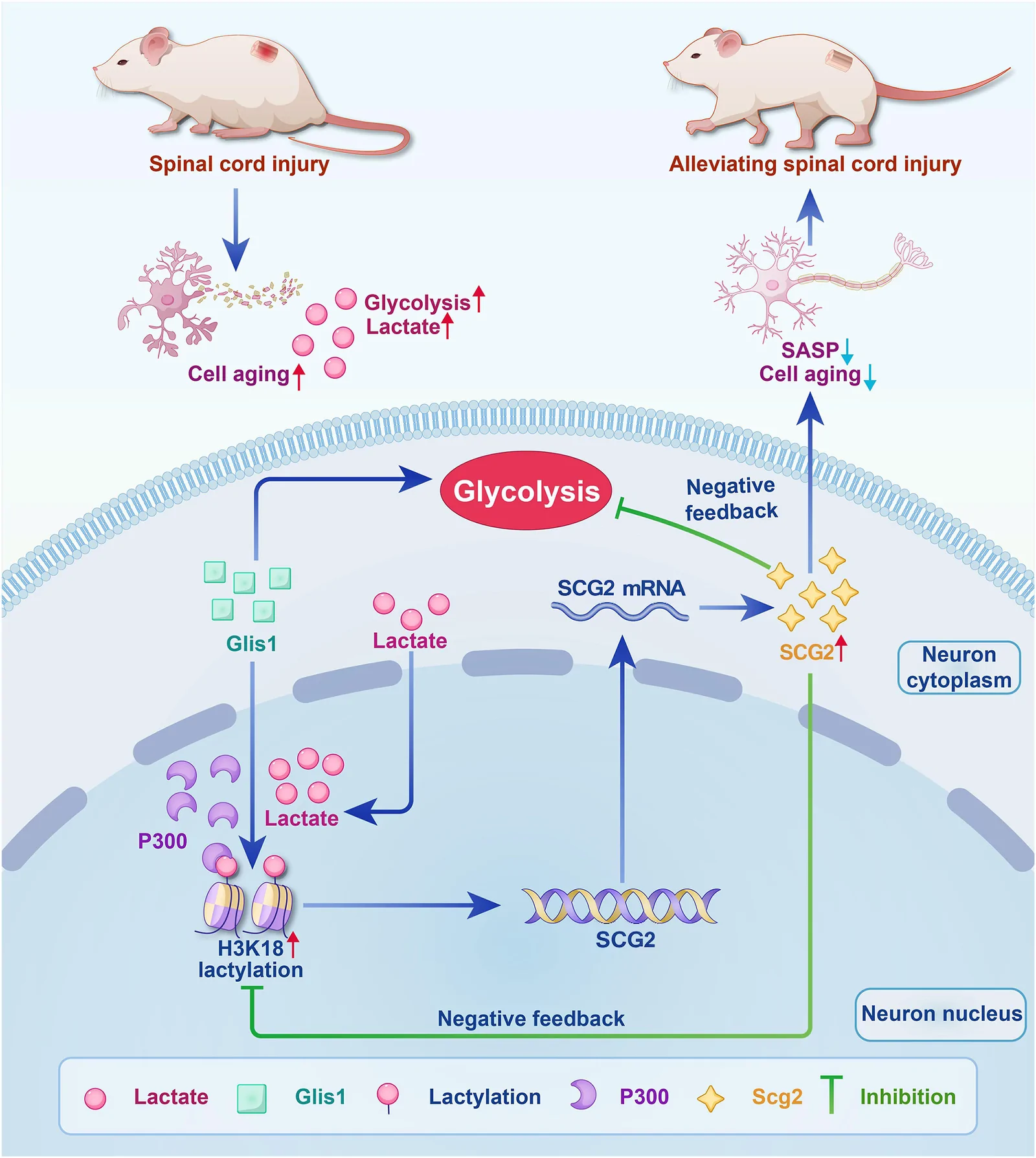
Glis1 initiates H3K18 lactylation via p300 and regulates the expression of downstream SCG2. The negative feedback loop among the expression of SCG2, glycolytic metabolism, and H3K18la modification undergoes metabolic reprogramming, thereby suppressing neuronal senescence.

## CRediT authorship contribution statement

**Dachuan Li:** Writing – original draft. **Xiao Lu:** Writing – original draft. **Zhaoyang Gong:** Formal analysis. **Jianwei Wu:** Formal analysis. **Yuxuan Zhang:** Writing – review & editing, Supervision. **Jiongdong Wu:** Writing – review & editing, Supervision. **Chenpei Xu:** Supervision. **Xingyu Guo:** Supervision. **Guangyu Xu:** Supervision. **Haocheng Xu:** Supervision. **Shuo Yang:** Supervision. **Xinlei Xia:** Supervision. **Feizhou Lu:** Supervision. **Jianyuan Jiang:** Supervision. **Shihao Lu:** Supervision. **Junzhe Sheng:** Supervision. **Xiaosheng Ma:** Supervision. **Wei Zhu:** Supervision. **Fei Zou:** Project administration. **Chi Sun:** Project administration. **Xiaochuan Gu:** Project administration. **Yunzhi Guan:** Supervision, Project administration, Funding acquisition.

## Ethics declaration

All animal experiments were formally examined and approved by the Animal Protection and Utilization Committee of Fudan University (No. 202408016S).

## Data availability

The data that support the findings of this study are available from the corresponding author upon request.

## Funding

This work was supported by the Youth Talent Cultivation Program for Basic Research at Huashan Hospital (China) (No. 2025JC012); the 10.13039/501100001809National Natural Science Foundation of China, China (No. 82572808, 82272549, 82472505, 82472498); the National Key Research and Development Plan, Ministry of Science and Technology of the People's Republic of China (No. 2022YFC2407203); and the Youth Talent Project of 10.13039/501100010120Huashan Hospital (China) (No. 30302164006).

## Conflict of interests

Each author certifies that he or she has no commercial associations (e.g., consultancies, stock ownership, equity interest, patent/licensing arrangements) that might pose a conflict of interest in connection with the submitted article.
