# Supplementary material for: Glis1 regulates metabolic programming by promoting H3K18 lactylation to alleviate neuronal senescence following spinal cord injury
Source: Genes Dis. 2026 Jul 23;14(2):102382. doi: 10.1016/j.gendis.2026.102382 (PMC13678048; doi:10.1016/j.gendis.2026.102382)
Supplement: Supplementary file 1 [file mmc1.docx]

Supplementary Materials for

**Glis1 regulates the metabolic programming by promoting H3K18 lactylation to alleviate neuronal senescence following spinal cord injury**

**Figure S1. Verification of neuronal cell identity in in vitro experiments.** (A) Observation and Identification of Spinal Cord Neurons. (a) Light microscopy of spinal cord neurons, showing neuronal cell bodies and axons. (b, c) Immunofluorescence identification of neurons. Red fluorescence indicates MAP2 protein expression, and blue fluorescence indicates DAPI-stained nuclei. (d) Merged image. (Scale bar: 20 μm). (B) Representative immunofluorescence images of primary spinal cord neurons in the Control group co-stained with NeuN (red, neuronal marker) Iba-1 (yellow, microglial marker) and GFAP (green, astroglial marker) (Scale bar: 20 μm). (C) Quantification of cell purity. (D, E) Representative immunofluorescence images and quantitative analysis of NeuN and p21 co-staining in the Control and LPS groups. (Scale bar: 20 μm). (F, G) Representative immunofluorescence images and quantitative analysis showing the co-localization of NeuN and TLR4 in the Control and LPS groups. (Scale bar: 20 μm). (H, I) Representative double immunofluorescence images and the quantification of the colocalization of Pan-Kla (green) and H3K18la (yellow) with the neuronal marker NeuN (red) in the neuronal cells in the Control and LPS groups. (Scale bar: 20 μm). （The relative contents were calculated using Image J software. n=3. All the data are expressed as means ± SD, one way ANOVA followed by Tukey's post hoc test was applied. ^#^P<0.05, ^##^P<0.01, ^###^P <0.001, ^####^P <0.0001, vs. Control; *P<0.05, **P<0.01, ***P <0.001, ****P <0.0001, vs. Iba-1; ^&^P<0.05, ^&&^P<0.01, ^&&&^P <0.001, ^&&&&^P <0.0001, vs. GFAP. ）

**A**

**B**

**C**

**D**

**Figure S2. Proteomic profiling and validation of Glis1-mediated regulation of SCG2.** (A) Volcano plots of differentially expressed proteins (DEPs) among different groups.(B) GSEA reveals significant enrichment of upregulated angiogenesis, glycolysis, and pro-peripheral neurite growth signaling pathways, as well as downregulated cell senescence signaling pathways.(C) PCR detects the mRNA expression level of SCG2 after treatment with LPS and pc-Glis1 or without.(D) PCR detects the mRNA expression level of SCG2 with or without the use of OXAMATE. （n=3. All the data are expressed as means ± SD, one or two- way ANOVA followed by Tukey's post hoc test was applied *P<0.05, **P<0.01, ***P <0.001, ****P <0.0001, vs. Control；^#^P< 0.05, ^##^P < 0.01, ^###^P < 0.001, ^####^p <0.0001, vs. LPS）

**Figure S3. Temporal profiling of pan-histone lactylation and H3K18 lactylation in neurons after spinal cord injury.** (A-B) Western blot analysis of the Pan histone lactylation and H3k18 lactylation levels in the spinal cord at Sham, and 3, 7, 14, and 28 dpi (n=3). (C-D) Representative images of Pan Kla , H3K18la co-stained with NeuN at Sham, 3, 7, 14, and 28 dpi in vivo (Scale bar: 20 μm, n=3). (The relative contents were calculated using Image J software. All the data are expressed as means ± SD, one or two- way ANOVA followed by Tukey's post hoc test was applied *P<0.05, **P<0.01, ***P <0.001, ****P <0.0001, vs. Sham.)

**Conditional knockout（CKO） mice**

Glis1 Nestin-CKO conditional knockout mice were generated by crossing the floxed Glis1 allele (Glis1^f/f^) mice with Nestin-Cre transgenic mice (#003771, The Jackson Laboratory), which expresses Cre recombinase in neural stem cells under the control of the Nestin promoter and conditional knockout genes in neural stem cells and as well as their derivatives including neurons[1]. Glis1^f/f^ mice were purchased from Cyagen Biosciences Co., Ltd. (Suzhou, China). Briefly, in the construct, exon 3 of mouse Glis1 (Gene ID:230587) is flanked by loxp sites for Cre-mediated deletion. Glis1 was functionally knocked out in Nestin-cre expressing cells after crossing Nestin-cre mice with loxp knock-in mice, resulting in a critical exon deletion and a frame shift. loxp-Glis1 mice were crossed with Nes-cre tool mice, and mice carrying both loxp-Glis1 and Cre genes were selected through multiple generations. Specifically, tamoxifen (#T5648, Millipore Sigma) was dissolved in corn oil at a concentration of 20 mg/ml and injected intraperitoneally into adult mice carrying loxp-Glis1 and Cre genes to activate the cre recombinase and cleave Glis1 at the loxp site to knock out Glis1 in neurons[2]. Expression of Cre recombinase in neuronal cells of these mice resulted in conditional deletion of Glis1. For the subsequent experimental procedures and methods of CKO mice, please refer to the Materials and Methods section of the text.

**Figure S4. Generation and characterization of Glis1 Nestin-CKO mice and the effects of neural Glis1 deletion after spinal cord injury.** (A-B) Diagrams showing the strategy used to generate Glis1 Nestin-CKO mice. (C) Genotyping of Glis1Nestin-CKO mice. (D) Western blot analysis of Glis1 expression in the spinal cords and various brain regions of Glis1-KO and Glis1Nestin-CKO mice. (E) Quantitative analysis of the relative Glis1 levels (n=3). (F) Western blot detection of glycolysis-related genes, H3K18la, SCG2 and senescence markers in each group and the quantitative results (n=3). (G-I) Representative images of expression levels of (G) NF, (H) GFAP and (I) SA-β-galdetected by immunofluorescence (Scale bar: 20 μm). (J) Quantitative analysis of the relative levels (n=3). (K-L) Representative (K) hematoxylin and eosin (H&E) and (L) Nissl staining of spinal cord tissues in each group (Nissl bodies were indicated by red triangular arrows) (n = 3). (M) BMS scores of different groups at 3, 7, 14, 21, and 28 days after SCI or sham surgery (n = 10). (The relative contents were calculated using Image J software. All the data are expressed as means ± SD, one or two- way ANOVA followed by Tukey's post hoc test was applied *P<0.05, **P<0.01, ***P <0.001, ****P <0.0001, vs. Glis1-KO; ^#^P<0.05, ^##^P<0.01, ^###^P <0.001, ^####^P <0.0001, vs. SCI-WT.)

**Figure S5. Validation of the Glis1–SCG2 regulatory axis and its effect on HUVEC migration after LPS treatment.** (A) Western blot analysis of sh-Glis1 expression. (B) Western blot analysis of Glis1 expression at Control, and 2 h, 8 h, 12 h, and 24 h after LPS treatment (n=3). (C) Chip analysis of the binding of the SCG2 promoter after LPS treatment (n=3). (D) The SCG2 overexpression vector with exogenous Flag was constructed. Western blot detection of endogenous SCG2 in each group and the quantitative results (n=3). (E) Migration assays of HUVECs cultured in neuronal CM following LPS treatment and SCG2 overexpression (Scale bar: 20 μm, n=3). (The relative contents were calculated using Image J software. All the data are expressed as means ± SD, one or two- way ANOVA followed by Tukey‘s post hoc test was applied ^P<0.05, ^^P<0.01, ^^^P <0.001, ^^^^P <0.0001, vs. sh-NC; *P<0.05, **P<0.01, ***P <0.001, ****P <0.0001, vs. Control；^#^P< 0.05, ^##^P < 0.01, ^###^P < 0.001, ^####^p <0.0001, vs. LPS；^&^P <0.05, ^&&^P < 0.01, ^&&&^P < 0.001,^&&&&^P < 0.0001, vs. LPS(12h),；^$^P <0.05, ^$$^P < 0.01, ^$$$^P < 0.001,^$$$$^P < 0.0001, vs. LPS+pc-Glis1.)

**Figure S6. Glis1 modulates spinal cord lesion severity and neuronal H3K18 lactylation-associated senescence after injury.** (A) PCR detects the mRNA expression level of Glis1 after treatment with LPS and si-Glis1 or pc-Glis1. (B) Quantification of the extent of spinal cord injury on MRI. Proportion of spinal cord high signal within 2mm of spinal cord injury site. (C) Representative images and the quantification of the expression level of NeuN with both H3K18la and p21 in spinal cord tissues detected by co-immunofluorescence. (Scale bar: 250 and 25 μm). (n=3, The relative contents were calculated using Image J software. All the data are expressed as means ± SD, one or two- way ANOVA followed by Tukey's post hoc test was applied &P<0.05, &&P<0.01, &&&P <0.001, &&&&P <0.0001, vs. LPS+si-Glis1; #P<0.05, ##P<0.01, ###P <0.001, ####P <0.0001, vs. LPS or SCI.)

**References**

[1]	F. Tronche, C. Kellendonk, O. Kretz, P. Gass, K. Anlag, P. C. Orban, R. Bock, R. Klein, G. Schütz, *Nat Genet* **1999**, *23* (1), 99, <https://doi.org/10.1038/12703>.

[2]	Y. Huang, H. Chen, D. Jin, S. R. Chen, H. L. Pan, *J Neurosci* **2023**, *43* (21), 3933, <https://doi.org/10.1523/jneurosci.0183-23.2023>.

**Table S1. Antibodies used in this study.**

| **Name** | **Manufacturer** | **Item No.** | **Ratio of dilution** |
| --- | --- | --- | --- |
| mouse anti- p53(WB) | Proteintech | 60283-2-Ig | 1:3000 |
| rabbit anti- p21(WB) | Abcam | ab109199 | 1:1000 |
| rabbit anti-p16 (WB) | Abcam | ab51243, | 1:2000 |
| rabbit anti-γH2AX (WB) | CST | #9718 | 1:500 |
| rabbit anti- IL-6 (WB) | affbiotech | DF6087 | 1:500 |
| rabbit anti-CCL2 (WB) | affbiotech | DF7577 | 1:500 |
| rabbit anti-Histone H3 (WB) | Proteomics | PTM-1406RM | 1:1000 |
| rabbit anti-Pankla (WB) | CST | #4499 | 1:1000 |
| rabbit anti-H3K18la (WB) | Proteomics | PTM-1401RM | 1:500 |
| rabbit anti-H3K9la(WB) | Proteomics | PTM-1419RM | 1:500 |
| rabbit anti-H4K8la (WB) | Proteomics | PTM-1415RM | 1:1000 |
| rabbit anti-H4K5la (WB) | Proteomics | PTM-1409 | 1:2000 |
| rabbit anti- H4K12la (WB) | Proteomics | PTM-1411RM | 1:1000 |
| mouse anti-Glis1 (WB) | Santa | sc-365453 | 1:500 |
| rabbit anti-c-Myc (WB) | Abcam | ab32072 | 1:1000 |
| rabbit anti-LDHA (WB) | CST | #2012 | 1:2000 |
| rabbit anti- GLUT1(WB) | Abcam | ab115730 | 1:2000 |
| rabbit anti-SCG2 (WB) | Proteintech | 20357-1-AP | 1:1000 |
| mouse anti-p300 (WB) | Santa | sc-32244 | 1:300 |
| rabbit anti- MOF (WB) | Santa | sc-32244 | 1:300 |
| mouse anti-GCN5 (WB) | Proteintech | 66575-1-Ig | 1:3000 |
| rabbit anti-TIP60 (WB) | Proteintech | 10827-1-AP | 1:1000 |
| rabbit anti-H3K18ac (WB) | Proteomics | PTM-160 | 1:1000 |
| mouse anti-H3K27ac (WB) | Proteomics | PTM-160 | 1:1000 |
| rabbit anti- DCX (WB) | Proteintech | 13925-1-AP | 1:2000 |
| rabbit anti- Tuj1(WB) | Abcam | ab68193 | 1:3000 |
| rabbit anti-α-Tubulin (WB) | Abcam | ab52866 | 1:10000 |
| rabbit anti-GAPDH (WB) | Abcam | ab181602 | 1:10000 |
|  |  |  |  |
| MAP2 antibody(IF) | Proteintech | 17490-1-AP | 1:200 |
| SA-β-gal antibody(IF) | Proteintech | 15518-1-AP | 1:200 |
| Actin antibody(IF) | Proteintech | 14651-1-AP | 1:400 |
| LAP2 antibody(IF) | Proteintech | 14651-1-AP | 1:100 |
| Pankla antibody(IF) | Proteomics | PTM-1401RM | 1:100 |
| H3k18la antibody(IF) | Proteomics | PTM-1406RM | 1:100 |
| p300 antibody(IF) | Santa | sc-32244 | 1:50 |
| SCG2 antibody(IF) | Proteintech | 20357-1-AP | 1:200 |
| Tuj1 antibody(IF) | Proteintech | 66375-1-Ig | 1:400 |
| CD31 antibody(IF) | Proteintech | 28083-1-AP | 1:400 |
| NF antibody(IF) | Abcam | ab215903 | 1:200 |
| NeuN antibody(IF) | Proteintech | 66836-1-Ig | 1:300 |
| Iba-1antibody(IF) | Abcam | ab178846 | 1:100 |
| GFAP antibody(IF) | Proteintech | 16825-1-AP | 1:600 |

**Table S2. Sequences of primers employed for Quantitative real-time polymerase chain reaction (qRT–PCR) in this study.**

| **Gene** | **Primer information** | | **Primer sequence（5`-3`）** | |
| --- | --- | --- | --- | --- |
| R-α-tubulin | NM_022298.1 | sense | | GCTGTGGTTGAGCCCTACAAT |
|  |  | antisense | | GTAGGTTGGGCGCTCAATGT |
| R-SCG2 | NM_022669 | sense | | GTCTTGAGTGAAGACGAGTGGAT |
|  |  | antisense | | GTCTCATAGTCATCAGGCGTGTC |
| Nestin-Cre | NC_000078 | sense | | TTGCTAAAGCGCTACATAGGA |
|  |  | antisense | | GCCTTATTGTGGAAGGACTG |
| Glis1-loxp | NC_000070 | sense | | GTTGGACTGTTACCTCCCTCCTGCCC |
|  |  | antisense | | ACATGCAAGGGGAGAGGGTGGGTCCC |
| R-Glis1 | XM_006240092.3 | sense | | GCACACTGTCGAGAGAAAGC |
|  |  | antisense | | TGTGGGTGGGTAAGGAGAGA |
